# The types and numbers of kinesins and dyneins transporting endocytic cargoes modulate their motility and response to tau

**DOI:** 10.1016/j.jbc.2024.107323

**Published:** 2024-04-25

**Authors:** Daniel Beaudet, Christopher L. Berger, Adam G. Hendricks

**Affiliations:** 1 Department of Bioengineering, McGill University, Montreal, Quebec, Canada; 2 Department of Molecular Physiology and Biophysics, University of Vermont, Burlington, Vermont, USA

**Keywords:** tau protein (tau), microtubule-associated protein (MAP), kinesin, dynein, organelle transport, *in vitro* reconstitution, optical tweezers, mathematical modeling

## Abstract

Organelles and vesicular cargoes are transported by teams of kinesin and dynein motors along microtubules. We isolated endocytic organelles from cells at different stages of maturation and reconstituted their motility along microtubules *in vitro*. We asked how the sets of motors transporting a cargo determine its motility and response to the microtubule-associated protein tau. Here, we find that phagosomes move in both directions along microtubules, but the directional bias changes during maturation. Early phagosomes exhibit retrograde-biased transport while late phagosomes are directionally unbiased. Correspondingly, early and late phagosomes are bound by different numbers and combinations of kinesins-1, -2, -3, and dynein. Tau stabilizes microtubules and directs transport within neurons. While single-molecule studies show that tau differentially regulates the motility of kinesins and dynein *in vitro*, less is known about its role in modulating the trafficking of endogenous cargoes transported by their native teams of motors. Previous studies showed that tau preferentially inhibits kinesin motors, which biases late phagosome transport towards the microtubule minus-end. Here, we show that tau strongly inhibits long-range, dynein-mediated motility of early phagosomes. Tau reduces forces generated by teams of dynein motors on early phagosomes and accelerates dynein unbinding under load. Thus, cargoes differentially respond to tau, where dynein complexes on early phagosomes are more sensitive to tau inhibition than those on late phagosomes. Mathematical modeling further explains how small changes in the number of kinesins and dynein on cargoes impact the net directionality but also that cargoes with different sets of motors respond differently to tau.

Motor-mediated transport is essential for the targeted delivery and function of organelles and vesicular cargoes in cells. Kinesins-1, -2, and -3 are the principal plus-end–directed microtubule-based motors that drive anterograde transport, while cytoplasmic dynein drives minus-end–directed retrograde transport (1, 2). Cargoes bound simultaneously by kinesin and dynein move bidirectionally along microtubules, which enables them to navigate around obstacles and achieve long-range targeted trafficking throughout the crowded cellular environment (3, 4, 5, 6). Intracellular cargoes exhibit diverse motility due to differences in their sets of kinesins and dynein motors and the regulatory mechanisms that coordinate their opposing activity. For instance, early endosomes are bound by kinesin-1, kinesin-3, and dynein and typically exhibit short bursts of unidirectional motility (7, 8, 9, 10). In filamentous fungi, early endosomes are driven by tightly-bound kinesin-3 motors, and the transient binding of dynein causes their directionality to switch between anterograde and retrograde transport (11), whereas late endosomes and lysosomes that simultaneously associate with members of the kinesin-1, -2, -3 families and dynein exhibit robust bidirectional transport (7, 9, 12, 13, 14). Multiple types of kinesins on the same cargo could be required for different organelle functions or target organelles to different destinations by controlling the activity of specific kinesins or through their selective transport along microtubules with different posttranslational modifications (14, 15). Although many kinesin–cargo interactions have been identified (16, 17, 18), determining the full complement of native motors on most endogenous cargoes and how these sets of motors are regulated to achieve targeted trafficking remains a challenge.

Microtubule tracks spatially regulate trafficking in cells. Microtubule-associated proteins (MAPs) organize the cytoskeleton by controlling the polarity, bundling, and stability of microtubules, as well as the interactions and movement of motor proteins along them (6, 19, 20, 21, 22, 23, 24, 25, 26, 27, 28). Several MAPs regulate the movement of motor proteins. One of the most studied MAPs is tau, which is implicated in Alzheimer’s Disease and related neurodegenerative diseases collectively known as tauopathies. Pathogenic forms of tau perturb the organization of the axonal cytoskeleton and lead to synaptic disfunction and neuronal dystrophy (28). Despite the link between aberrant tau regulation and neuropathology, it remains unclear what role tau plays in regulating axonal transport in healthy neurons and how defects lead to neurodegeneration.

Single-molecule studies show that microtubule motors have varying sensitivities to tau. Tau strongly reduces the processivity and landing rates of kinesin-1 and kinesin-3 along microtubules *in vitro* (26, 29, 30, 31, 32). In contrast, kinesin-2 is less processive but is better able to navigate around tau due to its longer more flexible neck linker domain (33, 34). Dynein is also less sensitive than kinesin-1 and kinesin-3 as it often pauses then proceeds to pass-through tau along microtubules (30, 35, 36). In addition, there are multiple isoforms of tau expressed throughout the adult brain that have different impacts on the motility of motor proteins. The 3RS-tau isoform strongly inhibits kinesin-1 and dynein processivity compared to 4RL-tau (29, 30, 31, 35). This is likely because the 3RS-tau isoform binds more statically along microtubules and has a higher propensity to form patches than 4RL-tau (37, 38). Tau patch formation is a potential regulatory mechanism to selectively govern the accessibility of the microtubule surface for motors and other MAPs (36, 39). Conversely, patches or larger-order tau complexes that form along microtubules could also act as precursors to tau-aggregates and neurofibrillary tangles found in neurodegenerative disease (40). Taken together, these findings suggest that tau functions as a selective barrier along the microtubule lattice that allows some types of motors to pass through while impeding others. Although these studies provide detailed biophysical characterizations of tau’s impact on individual motors, endogenous cargoes are often transported by teams of multiple kinesins and dyneins, and it is less clear how these heterogenous teams of motors are regulated by tau.

MAPs decorate the microtubules that serve as tracks for many types of cargoes, leading us to ask how MAPs might target specific cargoes to different destinations in the cell. To address this question, we characterized the sets of motors bound to endocytic cargoes and used *in vitro* reconstitution to test the impact of 3RS-tau on their motility along microtubules. We used isolated phagosomes to study tau-mediated transport due to their suitability for *in vitro* reconstitution motility assays, optical trapping for force measurements, and quantification by immunofluorescence to determine the type and number of motors bound to them. We made a quantitative comparison of the sets of motors on different cargoes and found that differences in the number and types of motors determine their motility and regulation by MAPs. Phagosomes are transported bidirectionally throughout maturation. Early phagosomes (EPs) move with a slight minus-end–directed bias compared to late phagosomes (LPs) that move equally in both directions. We found that the numbers of kinesin-1 and kinesin-3 motors on EPs and LPs were similar, but EPs had fewer dynein and kinesin-2 than LPs. In addition, EPs were more heterogenous and bound by fewer motors in total than LPs. Our previous work showed that isolated LPs move bidirectionally *in vitro* and tau biases their transport towards the minus-end of microtubules (6). Here, we show that tau more strongly inhibits retrograde EP motility. Tau reduces the number of long-range minus-end–directed transport events but has less of an inhibitory effect on short minus-end– or plus-end–directed transport. In addition, we show that tau reduces the overall magnitude of forces exerted by EPs with the highest reduction of forces generated by teams of multiple dynein motors. Our results combined with mathematical modeling show that differences in the number and type of motors present on cargoes determine their movement and response to tau. Thus, tau directs the transport of different cargoes by selectively tuning the motility and forces exerted by kinesin and dynein teams.

## Results

### EPs and LPs move differently and are transported by different sets of motors

**Figure 1:**
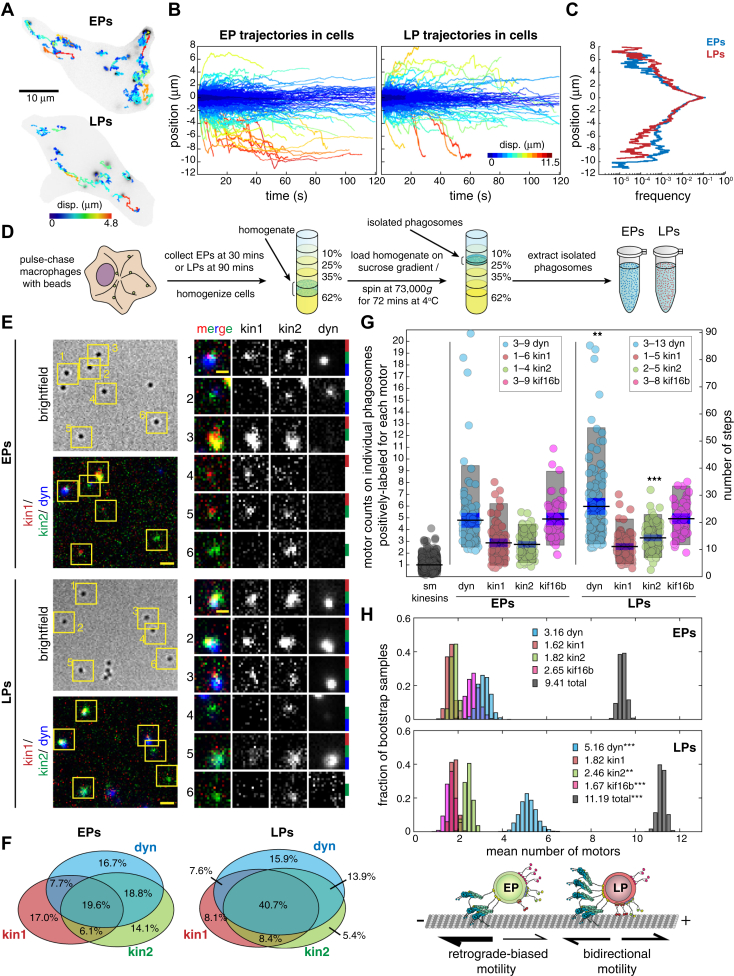
**EPs and LPs exhibit diverse motility and are transported by different sets of motors.***A*, images of J774A.1 mouse macrophage treated with 200 nm latex fluorescent beads overlaid with positional tracking data, color-coded by displacement (TrackMate). Time-lapse images were acquired at 30 min or 90 min after bead uptake to visualize bead-containing early phagosomes (EPs, *top cell*) or late phagosomes (LPs, *bottom cell*), respectively. *B*, the plots show the position of EPs (2211 trajectories from nine cells) and LPs (1579 trajectories from seven cells) towards the cell center (minus-end direction) or the cell periphery (plus-end direction). *C*, a plot shows the frequency of plus-end and minus-end EP and LP trajectories as a function of position. EPs more often travel longer distances towards the cell center than LPs (towards cell periphery, *p* < 0.05; towards cell center, *p* < 0.0001 by two-sample Kolmogorov-Smirnov (K-S) test). *D*, a schematic shows the steps to isolate phagosomes from cells. Isolated phagosomes migrate to a thin band between the 10% and 25% sucrose solutions. *E*, images show isolated EPs (*top panel*) and LPs (*bottom panel*) immunolabeled for kinesin-1 (*red*), kinesin-2 (*green*), and dynein (*blue*). Only single phagosomes were considered for analysis. On the right, zoomed in ROIs show examples of the combinations of motors on individual phagosomes. Colored bars on the right indicate the motors present for each example. Scale bars are 2 μm or 1 μm for zoomed-in ROIs. *F*, venn diagrams show the mean % of cargo bound by single motors or combinations of motors (EPs, *n* = 230; LPs, *n* = 239 from three independent phagosome isolations). *G*, stepwise photobleaching analysis of Alexa647 immunolabeled motors was used to estimate the number of motors that associate with EPs and LPs. A plot shows the number of photobleaching steps and corresponding number of kinesin-1, kinesin-2, kif16b, and dynein on EPs and LPs, mean values (*black lines*), SEM (*blue bars*), and 90% confidence intervals (*gray bars*). The number of steps to photobleach single motors was estimated by photobleaching Alexa647 immunolabeled recombinant kinesin-1 (kif5c) and -2 (kif3a/b) or kif16b extracted from cell lysate (*n* > 1000). The step counts indicate that there were 1 to 6 kinesin-1 on EPs (*n* = 79) and 1 to 5 kinesin-1 on LPs (*n* = 82), 1 to 4 kinesin-2 on EPs (*n* = 82) and 2 to 5 kinesin-2 on LPs (*n* = 83), 3 to 9 kinesin-3 on EPs (*n* = 51) and 3 to 8 kinesin-3 on LPs (*n* = 49), and 3 to 9 dynein on EPs (*n* = 101) and 3 to 13 dynein on LPs (*n* = 107) from three independent phagosome isolations. *H*, histograms show the mean number of each motor and predicted total number of motors on individual EPs and LPs (see Experimental procedures). Bootstrapping was performed to resample the data sets 1000 times to determine the means and test the statistical significance of the mean number of each type of motor and total motors on EPs and LPs. The schematic depicts the correlation between the mean sets of motors and the net motility of each cargo. (∗∗*p* < 0.01, ∗∗∗*p* < 0.0001).

We first sought to determine how the sets of motors that associate with different cargoes correlate with their motility. To test this, we analyzed phagosomes at different stages of maturation. Latex bead-containing phagosomes are a powerful system to examine how the membrane composition, biochemical properties, and ensembles of motors transporting endocytic organelles change in response to maturation and fusion events (9, 41, 42, 43, 44, 45, 46). Here, we characterized the motility and quantified the sets of motors on EPs and LPs from mouse J774A.1 macrophage. To image phagosome motility, cells were treated with 200 nm fluorescent latex beads and the movement of bead-containing phagosomes was monitored using total internal reflection fluorescence (TIRF) microscopy. Time lapse images of EPs were recorded at 30 min after bead uptake and LPs were recorded at 90 min, which is consistent with time points previously shown to coincide with the biochemical markers of early and late stages of phagosome maturation in mouse macrophage (41, 42, 43, 44). First, we compared the trajectories of EPs and LPs using TrackMate (47) (Fig. 1*A*) and plotted their positions to show directionality towards the cell center (microtubule minus-end) or towards the cell periphery (microtubule plus-end) (Fig. 1*B*). We examined the orientation of microtubules in macrophage using SiR-tubulin to determine if transport directed towards the cell center and periphery was due to minus-end– and plus-end–directed motility, respectively. We found that most microtubules in macrophage emanate radially from the perinuclear region where 82.1% of microtubule plus-ends point towards the cell periphery, 12.6% point tangential to the cell center, and 5.3% point inwards, supporting that movement towards the cell center is mainly driven by minus-end–directed motors and movement towards the periphery is driven by plus-end–directed motors (Fig. S1*A*). EPs had a higher fraction of minus-end–directed trajectories than plus-end–directed trajectories and more frequently traveled farther distances towards the cell center compared to LPs that had less of a difference in the fraction of plus-end– and minus-end–directed trajectories (Figs. 1, *A*–*C* and S1*B*). Phagosomes display stationary, diffusive, and processive periods of motility (46, 48). Periods of processive motility were identified as segments of a trajectory between two reversal events with displacements greater than 150 nm, which is consistent with mean-squared displacement (MSD) analysis (6). Results show that EPs had a higher fraction of long, minus-end–directed processive runs and had fewer reversal events than LPs (Fig. S1, *C*–*E*). While most directed trafficking is thought to be driven along microtubule tracks by motor proteins, cargoes could also ‘hitchhike’ along dynamic microtubules to move around in cells. However, dynamic microtubules were previously shown to transport peripheral phagosomes at speeds below 0.1 μm/s compared to motor-driven events that transport phagosomes at faster speeds between 0.2 and 1.5 μm/s (49), which is consistent with the majority of the processive motility events observed in this study (Fig. S1*F*). Thus, our results suggest that most phagosomes first move towards the perinuclear region of the cell due to minus-end–directed biased transport, then transition to more directionally unbiased motility as they progress through maturation to potentially fuse with other organelles and adopt specialized functions (43, 44, 50).

The type and number of motor proteins that associate with cargoes governs their transport behavior. Previous studies showed that LPs are transported by teams of kinesin-1, kinesin-2, and dynein motors (6, 48, 49). However, it was not clear how the sets of motors bound to phagosomes change in response to maturation. To test this, we compared the number of kinesins-1, -2, -3, and dynein on EPs and LPs. First, we isolated EPs and LPs from cells in parallel and performed 3-color immunofluorescence imaging to detect the combination of motors on individual phagosomes. EPs and LPs were isolated from macrophages at 30 or 90 min after bead uptake and purified on a sucrose density gradient (Fig. 1*D*). The largest fraction of LPs (40.7%) was positive for kinesin-1, kinesin-2, and dynein (Fig. 1, *E* and *F*), in agreement with previous results (6). In contrast, the motor sets associated with EPs were more diverse and a higher fraction of EPs were found to have only one type of motor compared to LPs (Fig. 1, *E* and *F*). We also probed for kinesin-3 motors on phagosomes. We tested for kinesin-3 motors separately due to limitations in the number of fluorophores that can be used to detect multiple motors within the same experiment. EPs and LPs were co-immunolabeled for dynein and the kinesin-3 motors kif16b and kif1b (Fig. S1, *G* and *H*), which have been found to transport similar endocytic organelles (8, 12, 51). We found that a higher fraction of EPs was positive for kif16b than LPs. The detection levels for kif1b were comparable to nonspecific binding controls (Fig. S1, I and J).

Using stepwise photobleaching analysis, we estimated the number of kinesins-1, -2, -3, and dynein on EPs and LPs. Isolated phagosomes were immunolabeled for each motor with Alexa647 antibody separately and imaged using epifluorescence illumination until the fluorescence signal was completely photobleached (Fig. S2*A*). A step-finding algorithm was applied to each intensity trace and used to measure the size and number of steps (Fig. S2, *B*–*F*) (6, 52). Single recombinant kinesin-1 (kif5c) and -2 (kif3a/b) and kif16b from cell lysate were also immunolabeled with Alexa647 and imaged under similar conditions, but instead using TIRF to observe clear photobleaching steps, to determine the number of fluorophores bound to a single motor (Fig. S2, *A*–*C*). The mean number of steps to photobleach a single motor was 4.28 steps. However, the number of steps ranged from 1 to 10, indicating that labeling efficiency or aggregation of single motors could be potential sources of error (Fig. S2*C*). For those phagosomes that were positively labeled, EPs and LPs had similar numbers of kinesin-1 and kinesin-3 but LPs were frequently associated with greater numbers of dynein and kinesin-2 motors (Fig. 1*G*), consistent with previous findings (5, 6, 53). To illustrate how a group of motors on a cargo changes as it responds to maturation, we estimated the number of kinesins-1, -2, -3 and dynein motors bound to individual EPs and LPs, taking into account the fraction of phagosomes where a given motor was not detected (see Experimental procedures). This approach enabled us to generate populations of simulated EPs and LPs that consisted of motor sets with specific numbers of each motor type found on cargoes at the frequencies that were observed experimentally. Our predictions indicate that ∼51% of LPs were bound by three or more different motors, compared to ∼39% of EPs (Fig. S1*K*). Further, the average individual EP was bound by fewer dynein and kinesin-2 than LPs but bound by similar numbers of kinesin-1 and kinesin-3 motors (Figs. 1*H* and S2, *G* and *H*). Thus, small changes in the number of motors bound to a cargo and therefore the number of motors that can engage the microtubule result in large changes in a cargo’s motility (Fig. 1*H*) (54, 55).

### Tau inhibits minus-end–directed EP motility

**Figure 2:**
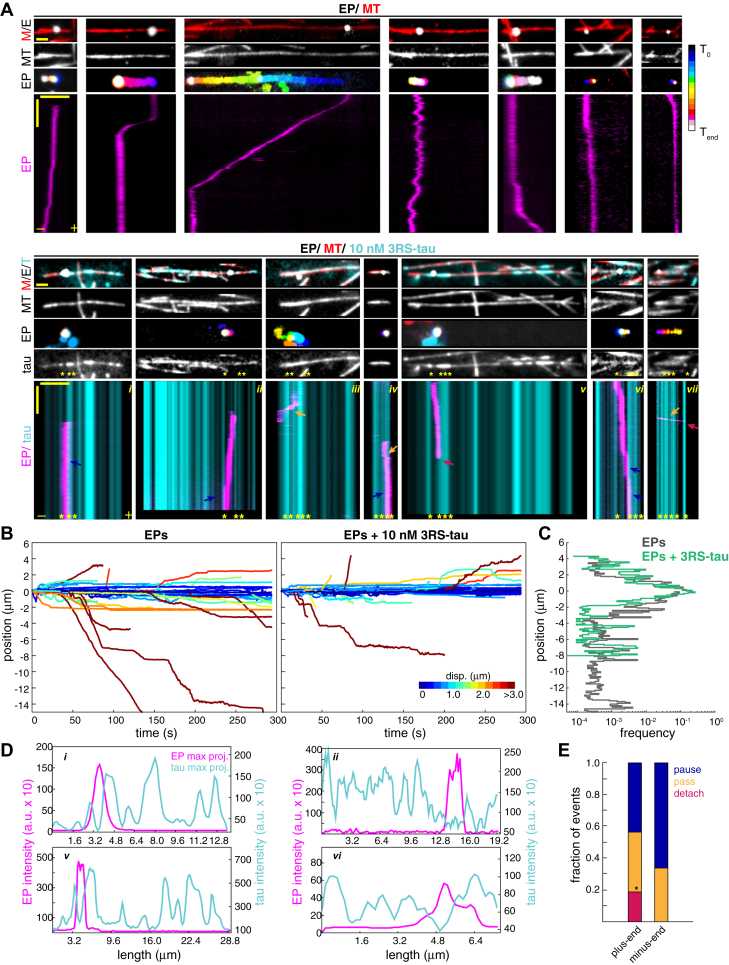
**Tau reduces minus-end–directed EP motility *in vitro*.***A*, early phagosomes (EPs) were extracted from cells and their motility was reconstituted *in vitro* along polarity-marked microtubules (bright segment; minus-end) ± 10 nM 3RS-tau. Images show microtubules (MT), tau, and max projections of EPs temporally color-coded. Kymographs show examples of unidirectional plus-end and minus-end transport and bidirectional transport ± tau. EPs often pause (*blue arrows*) and less frequently pass through (*orange arrows*) or detach from the microtubule (*magenta arrows*) when they encounter tau patches (*yellow asterisks*). Horizontal scale bars are 2 μm for images and 5 μm for kymographs, and the vertical scale bars for kymographs are 30 s. *B*, plots show trajectories of EPs directed towards the plus-end or minus-end of microtubules ± tau (control, *n* = 55 from 10 independent experiments; tau, *n* = 49 from eight independent experiments). Trajectories are color coded by displacement. *C*, a plot shows the frequency of plus-end and minus-end EP trajectories ± tau as a function of position. Tau reduces the frequency of EPs transported towards microtubule minus-end positions but does not impact the frequency of EPs transported towards plus-end positions (plus-end, *p* = 0.1438; minus-end, *p* < 0.0001 by K-S test). *D*, plots show linescans of the maximum projections of EPs and tau fluorescence signals along microtubules. Example plots of images *i, ii, v*, and *vi* from panel A show that EPs often move in areas of the microtubule where tau intensity is low. EP fluorescence intensity is shown on the *left* y-axis and tau fluorescence intensity is shown on the *right* y-axis. *E*, a bar graph shows the percentage of EPs that detach, pause, or pass-through tau patches (plus-end events, *n* = 16; minus-end events, *n* = 27). The data were analyzed using the Fisher’s exact test (∗*p* < 0.05).

The motor sets associated with EPs and LPs bear resemblance to those transporting native early endosomes, late endosomes, and lysosomes in cultured cells and neurons (7, 8, 9, 10, 11, 12, 13, 14, 15, 16, 17). However, it is difficult to examine the direct effects of tau on the motility of these cargoes due to the complexity and density of the native cellular environment. To overcome these challenges, we used *in vitro* reconstitution assays to investigate tau's effects on the motility of EPs, which are transported by different sets of motors and move differently than LPs. We previously showed that LPs move bidirectionally along microtubules *in vitro* and that tau biases motility towards the microtubule minus-end (6). Here, we tested the impact of tau on the motility of EPs under the same conditions. We reconstituted the motility of isolated EPs along taxol-stabilized, fluorescently labeled microtubules polymerized from bright GMPCPP seeds with or without the addition of 10 nM 3RS-tau. Time lapse recordings of EP motility events were imaged using TIRF microscopy and analyzed by subpixel tracking using 2D-Gaussian-fitting (Fluorescence Image Evaluation Software for Tracking and Analysis, FIESTA) (56). While EPs in cells display frequent reversals and bidirectional motility with a mild minus-end–directed bias (Figs. 1, *B* and *C* and S1, *A*–*D*), isolated EPs also exhibited bidirectional transport but had a much higher fraction of net motion directed toward the minus-end of microtubules and less frequently reversed directions (Figs. 2, *A*–*C* and S3, *A*–*C*). The lower reversal rate and higher degree of minus-end–directed biased motility observed *in vitro* compared to in cells is likely caused by EPs having fewer motors bound, reducing the probability that opposing motors will engage a single microtubule simultaneously. Whereas in cells, EPs have a higher probability of engaging several microtubules simultaneously due to the denser microtubule network, resulting in more frequent reversals. In addition, cellular signaling and other regulatory mechanisms are absent from the *in vitro* environment, where isolated EPs could be ‘locked’ in a configuration in which only a subset of motors are active, resulting in more frequent unidirectional transport. In the presence of 10 nM 3RS tau, the frequency of long-range minus-end–directed EP trajectories was reduced compared to shorter trajectories (<1 μm) or plus-end–directed trajectories, but no significant change occurred in the reversal rate (Figs. 2, *A*–*C* and S3, *B*–*D*). We also noted that the frequency of observable motility events with tau decreased approximately 2-fold, which is likely due to competition for available binding sites between motors and tau (27, 57). In support, linescans of tau and EP max projections show that EPs often bound and proceeded to move in areas of the microtubule where tau levels were low, indicating that tau reduces access of EPs to the microtubule surface (Fig. 2*D*). We also examined how tau impacts the motility of those EPs that bind and move along microtubules and found that 44% of plus-end– and 67% of minus-end–directed EPs paused when they encountered higher levels of tau, whereas 37% of plus-end– and 33% of minus-end–directed cargoes passed through tau (Fig. 2*E*). Furthermore, 19% of plus-end–directed cargoes detached from microtubules when they encountered tau compared to minus-end–directed cargoes that remained attached (Fig. 2*E*). These results are in agreement with recent studies that showed dynein-dynactin-BicD2N complexes are less sensitive to tau and often pass through or pause at tau patches compared to diffusive dynein complexes or kinesin-1 motors that are incapable of passing through tau or detach from the microtubule (30, 36, 39). Our findings further support a model in which tau acts as a selective barrier to control accessibility of the microtubule surface to different cargoes, as well as target their directional transport depending on the sets of motors engaged along them.

**Figure 3:**
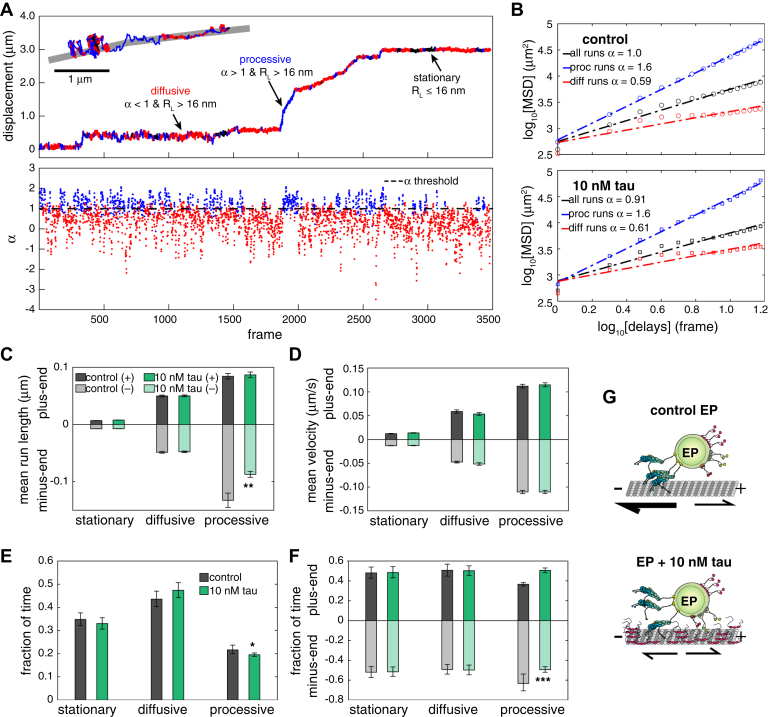
**Change-point analysis reveals that tau reduces EP minus-end–directed processive transport.***A*, plots show how change-point analysis identified stationary (*black*), diffusive (*red*), and processive (*blue*) periods of motility for each trajectory (*top graph*) by calculating local alpha (α) values using a rolling MSD with a sliding window (*bottom graph*). The inset (*top*, *left*) shows positional tracking data of an EP along a microtubule using FIESTA. The difference in the α-values of successive segments must be > 0.3 to be considered a change-point. Stationary runs were identified as the period of motility between two change-points with a run length (R_L_) ≤ 16 nm, which is the calculated mean tracking error (Fig. S4*A*), diffusive runs were categorized as runs with R_L_ > 16 nm and α ≤ 1, and processive runs were categorized as runs with R_L_ > 16 nm and α > 1. The trajectory is colored to show periods of stationary, diffusive, and processive motility. *B*, the graphs show log-log MSD of EP transport ± tau following change-point analysis. The α-values are shown for all runs, diffusive runs, and processive runs for control EPs (*top*) and EPs + tau (*bottom*). Bar graphs show (*C*) the mean run lengths and (*D*) mean velocities for stationary (ctl (+), *n* = 863; ctl (−), *n* = 895; tau (+), *n* = 791; tau (−), *n* = 856), diffusive (ctl (+), *n* = 629; ctl (−), *n* = 616; tau (+), *n* =680; tau (−), *n* = 660), and processive (ctl (+), *n* = 748; ctl (−), *n* = 923; tau (+), *n* = 885, tau (−), *n* = 831) runs identified by change point analysis ± tau. Error bars show SEM. *E*, a bar graph shows that tau does not significantly change the fraction of time of stationary and diffusive motility but slightly decreases processive motility. *F*, a bar graph shows the fraction of time of stationary, diffusive, and processive runs in the minus-end or plus-end directions ± tau. Error bars in (*E*) and (*F*) show 95% confidence intervals. Statistical significance and confidence intervals were determined by bootstrapping (Fig. S4, *C*–*F*). *G*, a cartoon schematic describes the impact of tau on EP motility. EP transport is biased towards the microtubule minus-end (*top*). Tau reduces the run lengths and frequency of minus-end directed runs (*bottom*), which causes EP motility to shift to shorter runs with an equal fraction of plus-end and minus-end directed events. (∗*p* < 0.05, ∗∗*p* < 0.001, ∗∗∗*p* < 0.0001). MSD, mean-squared displacement.

Isolated EPs exhibit periods of stationary, diffusive, and processive transport along microtubules *in vitro*. To determine the contribution of each mode of transport to their overall motility, we segmented trajectories into stationary, diffusive, and processive periods using change point analysis (Fig. 3*A*). Cargo trajectories were projected along the length of microtubules to track their on-axis position and directionality (Fig. 3*A*). The tracking uncertainty was determined to be 16 nm from the tracked positions of immotile phagosomes (Fig. S4*A*). Based on the MSD and tracking uncertainty, runs were considered stationary if the run length (R_L_) ≤ 16 nm, diffusive if R_L_ > 16 nm and α ≤ 1, and processive if R_L_ > 16 nm and α > 1 (Figs. 3*A* and S4*B*). The MSD for all runs with or without tau indicates that most EP motility was stationary or diffusive (control EPs α = 1.0 and EPs with tau α = 0.91) (Fig. 3*B*). The group of runs that were identified as processive by change point analysis had α-values that reflect motor-mediated transport (control EPs α = 1.6 and EPs with tau α = 1.6), whereas runs identified as stationary or diffusive had lower α-values (control EPs α = 0.59 and EPs with tau α = 0.61), indicative of constrained diffusion along the microtubule lattice (Fig. 3*B*). Tau did not change the mean lengths of stationary, diffusive, and plus-end–directed processive runs but reduced minus-end–directed processive runs (Fig. 3*C*), whereas the mean velocities for each category remained unchanged (Fig. 3*D*). EPs predominantly exhibit stationary or diffusive motility and only 22% of the time exhibit periods of processive movements (Fig. 3*E*). Tau did not drastically alter the fraction of time of stationary, diffusive, or processive motility but altered the net directionality of processive motility (Fig. 3, *E* and *F*). While stationary and diffusive runs were found to be directionally unbiased, 63% of processive transport was directed towards the minus-end of microtubules in the absence of tau compared to 49% when tau was present (Fig. 3*F*). Combined, these results show that EPs are directionally biased towards the minus-end of microtubules, and tau strongly inhibits minus-end–directed motility compared to its effects on plus-end–directed motility (Fig. 3*G*).

### Tau strongly inhibits long-range minus-end–directed processive runs

**Figure 4:**
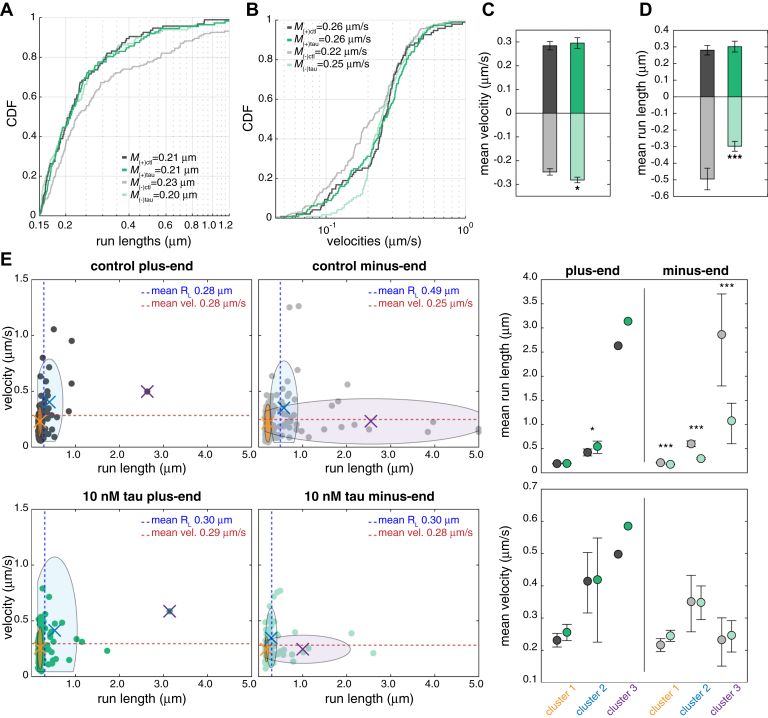
**Tau strongly inhibits long-range minus-end–directed processive runs.***A*, the cumulative distribution function (CDF) of run lengths of plus-end– and minus-end–directed processive runs ≥150 nm ± tau (95 plus-end and 162 minus-end control runs and 112 plus-end and 116 minus-end runs with tau). Tau decreases the number of long minus-end–directed processive runs but does not impact plus-end–directed runs. (plus-end runs, *p* = 0.9250; minus-end runs, *p* < 0.05). *B*, the CDF of the velocities of plus-end– and minus-end–directed processive runs ≥150 nm ± tau. Tau decreases the fraction of runs with slower velocities but has less impact on the fraction of runs with higher velocities or plus-end–directed runs (plus-end runs, *p* = 0.3453; minus-end runs, *p* < 0.0001). Median (M) run lengths and velocities are shown in (*A*) and (*B*). The K-S test was used to determine the statistical significance of tau’s effects on run lengths and velocities. Bar graphs show (*C*) the mean run lengths and (*D*) mean velocities of plus-end– and minus-end–directed processive runs ≥ 150 nm ± tau. Error bars indicate SEM. *E*, plots show velocities *versus* run lengths of plus-end– and minus-end–directed processive runs for control (*top*) and + tau (*bottom*). Clustering analysis was used to identify groups of runs by velocity and run length. Plus-end–directed runs fit within two clusters, whereas minus-end–directed runs fit within three clusters. Each colored (X) represents the mean of each cluster. On the *right*, plots show the mean run length and velocity of each cluster. Minus-end–directed runs with low velocities and longer run lengths are more strongly inhibited by tau than shorter faster runs or plus-end–directed runs. (∗*p* < 0.05, ∗∗∗*p* < 0.0001).

To dissect tau’s role in regulating long-range motor-mediated EP motility, we analyzed the run lengths and velocities of processive runs. Based on the MSD, we focused on processive runs with R_L_ ≥ 150 nm, since shorter runs classified as processive could be caused by directionally biased diffusion along the microtubule lattice (Fig. S4*B*). In addition, tau did not have any noticeable effects on the velocities or run lengths of stationary, diffusive, and processive runs with R_L_ < 150 nm (Fig. S5, *A*–*C*). The distributions of plus-end– and minus-end–directed processive runs with R_L_ ≥ 150 nm parsed by length closely followed an exponential decay where long runs were rarer than short runs but more frequently directed towards the microtubule minus-end (Fig. S5*D*). Tau reduced the number of long minus-end–directed processive runs, while short runs or plus-end–directed runs were unaffected (Figs. 4*A* and S5*D*). Minus-end–directed processive runs with lower velocities were also less frequent with tau, whereas plus-end–directed velocities did not change (Figs. 4*B* and S5*E*). With tau, the mean run length of minus-end–directed runs decreased from 0.49 μm to 0.30 μm and the mean velocity increased from 0.25 μm/s to 0.28 μm/s, while the mean run length and velocity of plus-end runs were not significantly affected (Figs. 4, *C* and *D*, S5, *D* and *E*). We used clustering analysis based on the relationship between velocity and run length to determine tau’s impact on plus-end– and minus-end–directed processive motility (Fig. 4*E*). Plus-end–directed runs fit within two distinct groups that proportionally increase in velocity and run length, whereas three groups of minus-end–directed runs were identified where the first and second groups increase proportionally by velocity and run length, but a third group consisted of runs with longer run lengths and slower velocities (Fig. 4*E*). Tau significantly reduced the number of runs within the group of slow, long-range minus-end–directed runs compared to its effects on the groups of faster, shorter minus-end runs or plus-end–directed runs (Fig. 4*E*). These results suggest that for minus-end–directed cargoes like EPs, tau acts as an obstacle on the microtubule that more strongly inhibits long dynein-driven runs compared to the relatively short and infrequent kinesin-driven runs.

### Tau reduces the forces generated by teams of multiple dyneins transporting EPs

**Figure 5:**
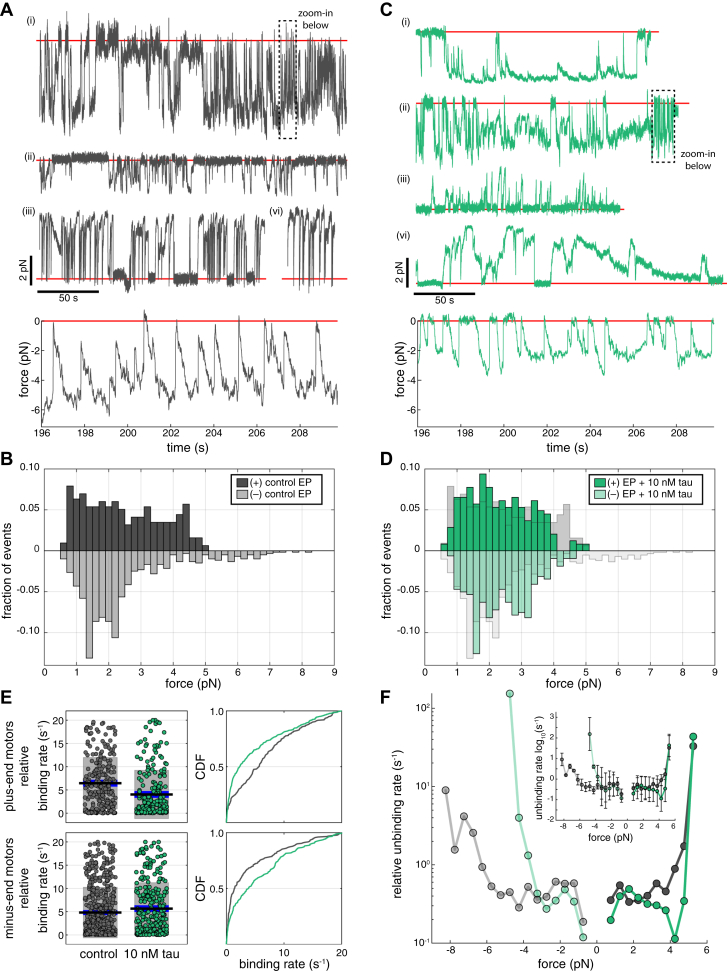
**Tau reduces the forces generated by teams of multiple dynein transporting EPs.***A* and *C*, force traces of EPs ± 10 nM tau were acquired at 2 kHz using an optical trap and median-filtered at 20 Hz. Force events were observed in both the minus-end direction (*i*, *ii*) and the plus-end direction (*iii*, *iv*) for (*A*) control EPs (*n* = 904 events from 64 recordings taken over 11 independent experiments) and (*C*) EPs + tau (*n* = 563 events from 54 recordings over 11 experiments). *Red lines* show the trap center (0 pN). *B* and *D*, histograms show the distribution of plus-end and minus-end force events with stall durations > 250 ms for (*B*) control EPs and (*D*) EPs + tau. Control EP force events were overlaid (*light gray bars*) on top of EP+ tau force events to show how tau reduced higher force events in both directions (plus-end forces, *p* < 0.05; minus-end forces, *p* < 0.001 by K-S test). *E*, plots show the relative binding rates calculated for plus-end motors (*top*) and minus-end motors (*bottom*). On the *right*, CDF plots show how tau influences the fraction of the relative binding rates of plus-end and minus-end motors (plus-end motors, *p* < 0.0001; minus-end motors, *p* < 0.05 by K-S test). *F*, a plot shows the force-dependent relative unbinding rates of minus-end– and plus-end–directed forces ± tau with stall times > 250 ms. The inset shows the unbinding rates on a log-scale y-axis. Error bars indicate SEM.

To understand how tau regulates the activity of teams of kinesins and dynein, we measured the forces generated by motors transporting EPs along microtubules using an optical trap. Previous studies showed that isolated LPs exert maximum forces of 9 to 12 pN *in vitro* and up to ∼20 pN in live cells (6, 24, 48, 58). The maximum forces generated by the sets of motors on isolated EPs reached stall forces of ∼8 pN directed towards the microtubule minus-end and ∼5 pN directed towards the microtubule plus-end (Figs. 5, *A* and *B*, S6, *A* and *B*), consistent with fewer motors being associated with EPs than LPs (Fig. 1*H*). EPs rapidly bind and unbind microtubules and exert unidirectional forces directed towards the plus-end or minus-end of microtubules with rare occurrences of phagosomes exerting bidirectional forces (9/63 = 0.1429, 95% CI = [0.0675, 0.2539] EPs showed bidirectional forces) (Fig. 5*A*). Consistent with their motility, EPs had a higher fraction of minus-end–directed events compared to plus-end–directed events (Figs. 3*F* and S6*C*). Plus-end events occurred over a range of forces with forces above ∼4 pN occurring less frequently than lower force events (Figs. 5*B* and S6*A*). The higher forces observed are consistent with the stall forces of a single kinesin-1 or kinesin-2 motor (59), while the higher fraction of lower forces between ∼1 and 3 pN indicate that kinesins often detach from the microtubule before maximum stall forces are reached (Fig. 5*B*). Alternatively, low-force events might also be driven by kinesin-3 motors (60). Minus-end forces frequently had lower magnitudes that ranged between ∼1 and 3 pN, consistent with sub-stall detachments or low-force stalls by a single dynein motor (6, 58, 61). There were also minus-end events with higher forces between ∼3 and 8 pN (Fig. 5*B*), indicative that 1 to 2 dynein motors might be complexed to dynactin and an adaptor such as BicD2, BicDR1, or HOOK3 (61, 62, 63, 64, 65) exerting forces along microtubules. In the presence of tau, force events directed towards the minus-end of microtubules occurred less frequently and the overall magnitude of forces in both directions decreased (Figs. 5, *C* and *D* and S6, *A*–*C*). Tau decreased the frequency of plus-end maximum stall forces above 4 pN, while the frequency of lower force events remained unchanged (Fig. 5, *C* and *D*). However, minus-end–directed forces > 5 pN were completely abolished with tau, followed by a moderate increase in the frequency of lower forces at ∼3 pN (Figs. 5*D* and S6*B*).

To compare the effects of tau on the rate at which kinesin and dynein bind to microtubules, we calculated the relative binding rates from the diffusive dwell times that occurred before each force event in the optical trap (Fig. S6*D*) (24). Tau decreased the relative binding rate of kinesins, whereas the binding rate of dynein increased slightly (Fig. 5*E*). In addition, we compared the effects of tau on the rate at which kinesins and dynein detach from microtubules, by using the method described by Berger *et al.* (66), which estimates the force-dependent unbinding rates from the probability that the motors detach within a given range of forces, with the simplifying assumption that the force dependence of the unbinding rate is exponential. We found that the unbinding rate for plus-end and minus-end force events with stall durations >250 ms increased exponentially with force (Fig. 5*F*). However, a higher rate of unbinding also occurred at lower forces with shorter stall times (<1 pN and >70 ms), which indicates that motors transporting EPs frequently unbind from the microtubule independent of force (Fig. 5*E*). Tau increased the unbinding rate of minus-end–directed forces, particularly at forces >3 pN (Fig. 5*F*). In contrast, tau did not affect plus-end forces between 0.5 and 4.0 pN or significantly alter the unbinding rate (Fig. 5*F*). The lack of an effect of tau on plus-end–directed forces is likely due to kinesin-driven forces being rare, consistent with the predominantly minus-end–directed motility of EPs (Figure 2, Figure 3, Figure 4). In contrast to bidirectional cargoes like LPs, the dynein teams on retrograde-biased cargoes are more sensitive to tau on microtubules and often detach before maximum forces are exerted.

### Differences in the sets of kinesins and dynein among populations of cargoes determines the effects of tau on their processive motility

**Figure 6:**
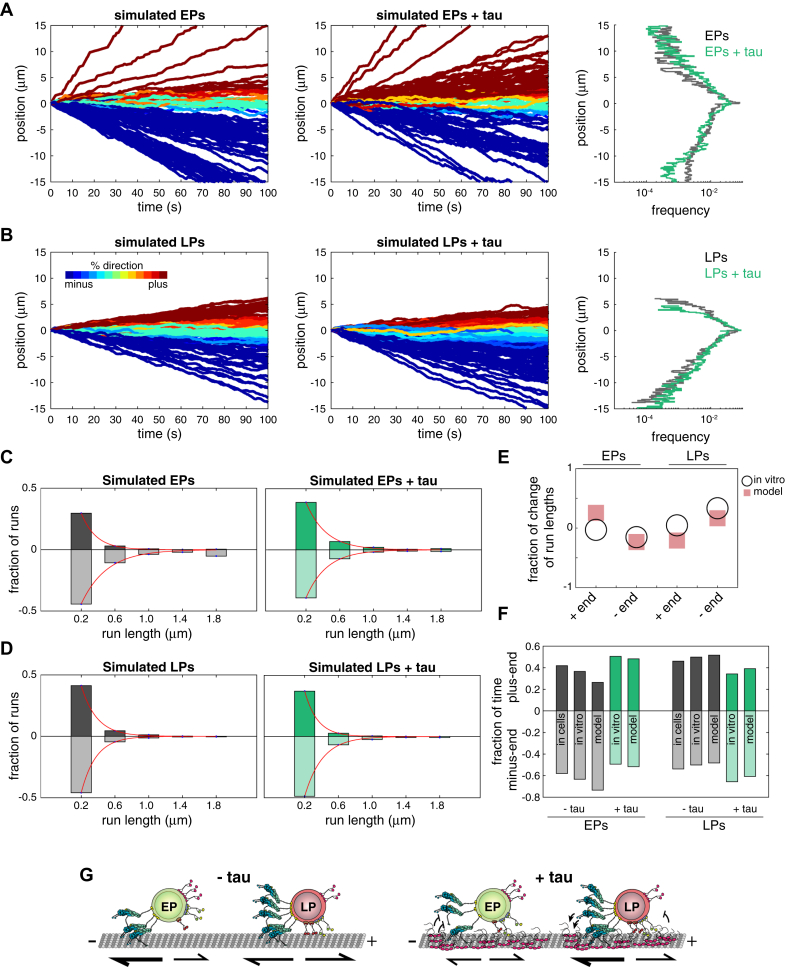
**Differences in the sets of kinesins and dynein among populations of cargoes determines the effects of tau on their processive motility.***A* and *B*, plots show simulated EP and LP trajectories directed towards the plus-end or minus-end of microtubules ± tau (for each condition, *n* = 100). Trajectories that recapitulate the processive periods of EP and LP transport were simulated using a stochastic tug-of-war model developed by Müller *et al.* (54) and extended to include cargoes transported by sets of kinesins −1, −2, −3, and dynein-dynactin-BicD2 (DDB). Modeling parameters from previous single-molecule studies, including the unbinding rate, binding rate, detachment force, stall force, and forward and backward velocities, were used to describe the motility of each motor (Table S1). Motor set heterogeneity among populations of EPs and LPs were incorporated in the model by random sampling along the CDF plots of each motor (Fig. S2*G*). Tau’s effects were modeled by increasing the unbinding rates of dynein, kinesin-1, and kinesin-3, consistent with previous studies (6, 33, 92). To the *right*, plots show the frequency of plus-end and minus-end EP and LP trajectories as a function of position. The frequencies of simulated EP and LP positions ± tau are different (plus-end EP positions, *p* < 0.05; minus-end EP positions, *p* < 0.0001; plus-end LP positions, *p* < 0.05; minus-end LP positions, *p* < 0.0001 by K-S test). *C* and *D*, histograms show the distributions of simulated EP and LP run lengths for plus-end– and minus-end–directed processive runs ± tau. Lines are single-exponential fits. Tau increased the lengths of EP plus-end and LP minus-end runs but decreased the lengths of EP minus-end and LP plus-end runs (EP plus-end, *p* < 0.01; EP minus-end, *p* < 0.0001; LP plus-end, *p* < 0.0001; LP minus-end, *p* < 0.05 by K-S test). *E*, the model closely approximates the effects of tau observed *in vitro*. The plot shows a comparison of the fraction of change of run lengths $\left(\frac{{{L\_r}_{\left\{tau\right\}}-\;L\_r}_{\left\{ctl\right\}\;}}{{L\_r}_{\left\{ctl\right\}}}\right)$ measured from the decay constants (L_r) from single-exponential fits of simulated and experimental runs. LP experimental *in vitro* data in panels (*D*) and (*F*) was obtained from Chaudhary *et al.* (6). *F*, a bar graph shows that the fraction of time of processive runs in the plus-end and minus-end direction for simulated EP and LP trajectories ± tau are consistent with experimental cell-based and *in vitro* results. *G*, a cartoon schematic shows how EPs and LPs move differently and have disparate responses to tau. While LPs are bidirectionally transported along microtubules, EPs more frequently move towards the minus-end of microtubules. In the presence of tau, LP transport is biased towards the microtubule minus-end, whereas EP long-range minus-end–directed transport is strongly inhibited.

Using mathematical modeling, we tested the hypothesis that the sets of motors bound to a cargo determine how it moves and responds to tau. We expanded the stochastic tug-of-war model used to describe bidirectional transport to include transport by sets of kinesins-1, -2, -3, and dynein-dynactin-BicD2 (DDB) (54). Single motor modeling parameters for kinesins-1, -2, -3, and DDB were obtained from previous studies (Table S1). To account for the heterogeneity of the sets of motors among phagosomes, we modeled transport using simulated populations of cargoes transported by different teams of motors. To do this, we randomly sampled the distributions of the numbers of motors associated with EPs and LPs that we measured and generated sets of motors where the number of motors of a given type on a single cargo were ±10% of its median probability of occurrence among the population (see Experimental procedures). Thus, simulated EPs were transported by sets of 1 to 3 DDB, 0 to 2 kinesin-1, 0 to 2 kinesin-2, and 0 to 3 kinesin-3 and LPs transported by sets of 4 to 5 DDB, 1 to 2 kinesin-1, 2 to 3 kinesin-2, and 0 kinesin-3 (Fig. 6, *A* and *B*) (6). To model the effects of tau on cargo transport, all single molecule motor parameters were constrained apart from the increased unbinding rates of DDB, kinesin-1, and kinesin-3 for EPs and LPs (Table S1). The change in the force-independent unbinding rates caused by tau were estimated from the run durations of processive runs for EPs (Fig. 4*A*) and LPs from Chaudhary *et al.* (6). To approximate the more inhibitory effect of tau on the minus-end–directed transport of EPs compared to LPs (6), the unbinding rate of DDB-transporting EPs was increased two-fold higher than the unbinding rate of DDB-transporting LPs. It is important to clarify that our modeling approach is limited as it only captures motor-mediated motility events, in which simulated cargoes are indefinitely driven along the microtubule by one or more motors. Thus, the simulations do not capture states where all the motors are not associated with the microtubule and the motility is dominated by diffusion (Figs. 1 and 2). Simulations show that tau reduced the lengths of individual runs within a trajectory and the frequency of minus-end–directed EP transport, whereas the run lengths and frequency of minus-end–directed LP transport increased (Fig. 6, *C* and *D*), which is consistent with experimental *in vitro* results obtained from Chaudhary *et al.* (6) and the present study (Fig. 6, *E* and *F*). Modeling supports that by altering the unbinding kinetics of kinesins-1, -3, and DDB, tau disparately effects endocytic cargoes transported by different sets of motors, which results in the strong inhibition of retrograde cargoes and the minus-end–biased transport of bidirectional cargoes (Fig. 6*G*). Thus, small changes in the number and type of motors present on cargoes causes drastic changes in their motility and how they respond to tau (Fig. 6*G*).

## Discussion

Our results show that tau has specific effects on the motility of each cargo, dependent on the sets of motors that drive their transport. EPs exhibit fewer long, minus-end–directed runs in the presence of tau (Figure 2, Figure 3, Figure 4), while tau enhances dynein-directed movement on LPs (6). We used *in vitro* reconstitution and single molecule approaches to characterize the transport machinery on endogenous cargoes and understand how tau impacts their transport. Isolated phagosomes provided an ideal system due to their optical properties and uniform size, enabling us to measure the motility of cargoes by their native sets of motor proteins and use an optical trap to probe for the biophysical effects of tau on their transport along microtubules in the absence of other cellular components. Combined, our data supports a model where tau acts as a selective barrier along microtubules to control the transport of cargoes bound by different sets of motors. We compared EPs and LPs and found that the types of motors associated with EPs are more variable than LPs and that EPs are bound by fewer dynein and kinesin-2 motors (Fig. 1, *E*–*H*). The differences in the sets of motors associated with them alter their motility and response to tau. LPs move bidirectionally with approximately equal fraction of directed motion towards the minus-end or plus-end of microtubules (Fig. 1, *B* and *C*), and we showed previously that their plus-end–directed forces decreased in the presence of tau, which concomitantly increased the processivity and opposing forces generated by dynein motors along microtubules (6). Here, we found that EPs exhibited a higher frequency of retrograde transport than LPs and more frequent long-range minus-end–directed runs *in vitro* (Figs. 1, *B* and *C*, 2, *A*–*C*, and 4, *A* and *E*). Tau reduced the processivity and maximum forces exerted by teams of dynein on EPs (Figs. 4 and 5), which decreased the frequency and travel distances of their minus-end–directed transport (Figure 2, Figure 3, Figure 4). Results from modeling in support of our experimental findings reveal how tau differentially regulates teams of motors transporting different cargoes (Fig. 6). Further, our results suggest that perturbations to tau would be expected to have disparate effects on different types of cargoes.

### What determines if a cargo moves unidirectionally or bidirectionally?

Our results are consistent with bidirectional motility resulting from a stochastic tug-of-war between opposing motors that are simultaneously bound to a cargo (54). Stochastic tug-of-war models predict that altering the number of kinesin and dynein motors that are bound to a cargo or adjusting their microtubule-binding kinetics determines the net directionality as well as the extent of bidirectional and unidirectional transport (5, 6, 54, 55, 67). We reason that EPs exhibit a higher fraction of unidirectional motility and directionally biased transport because they are typically bound by fewer copies of a single type of motor or combinations of two different motors, whereas LPs move bidirectionally because they are bound by multiple copies of kinesins-1, -2, -3, and dynein. Alternatively, EPs might be regulated such that opposing motors are not in an active state when they are isolated from cells. In support, our optical trapping data shows that EPs exert unidirectional forces that are comparable to the forces produced by a single kinesin or 1 to 2 dynein motors complexed with an activating adaptor protein such as BicD2, BicDR1, or HOOK3 (61, 62, 63, 64, 65) (Fig. 5, *A* and *B*). Whereas the magnitude of the forces exerted by LPs were previously shown to be approximately equal in both directions, which corresponds to forces produced by teams of up to three kinesin and ten dynein motors (6). However, recent studies suggest that this is an overestimate of the number of dynein motors engaged with microtubules that are opposing kinesins, since fewer active dynein–adaptor complexes, like those on endogenous cargoes, are required to exert higher forces than dynein alone (55, 61, 62). These results further indicate that only a subset of the total number of motors that are bound to cargoes are engaged with microtubules and exert force on EPs and LPs. Combined, this data suggests that as phagosomes mature, their total number of motors as well as the ratio of kinesin and dynein motors that engage the microtubule change, which potentiates their transition from retrograde-biased transport to directionally unbiased transport in cells.

While our data show that EPs and LPs exhibit different motility due to their association with different sets of kinesin and dynein motors, other factors likely modulate the activity of vesicle-bound motors. For instance, clustering of dynein was shown to enhance the processivity and forces exerted on cargo in *Dictyostelium* (46). Rai *et al.* (46) examined the motility of phagosomes isolated at ∼30 min, which is the same timepoint of EPs examined in this study. They observed similar long unidirectional runs, suggesting that motor clustering may be a conserved mechanism for the targeted long-range–directed transport of vesicles and organelles. Motors diffusively anchored to vesicle membranes has also been suggested to enable bidirectional transport, where during pauses, opposing motors that transiently interact with the microtubule compete in a tug-of-war resulting in directional switches (68). Differences in the lipid contents that alter the fluidity of membranes and diffusivity of motors on different cargoes may play a role in regulating the transport properties of different cargoes as well as their response to MAPs along microtubules (46, 69). Transient motor–cargo interactions were also shown to modulate early endosome transport in filamentous fungi, where binding of dynein outcompetes kinesin-3 motors causing a directional switch as they approach the hyphal tip, where pools of dynein are most concentrated (11). Similar mechanisms could ensure that phagosome trafficking is spatially and temporally controlled as they mature, where motors transiently interact with EPs and LPs or are reorganized on their membranes to bias their trafficking in different regions of the cell.

Adaptor proteins are functionally diverse and associate with different motors to control cargo transport. Adaptor–motor complexes recognize different cargoes through direct or indirect interactions with Rab GTPases and other membrane-associated regulatory effectors (70, 71, 72, 73, 74, 75, 76, 77). As phagosomes mature, the level of Rabs and other small GTPases change (44). These changes could also generate downstream effects that alter the type of adaptors and motors that associate with EPs and LPs, which in turn impact their motility (78, 79, 80, 81, 82). Adaptors also modulate the effects of tau on motor motility. TRAK1, which is a mitochondrial-specific adaptor protein, is an example of an adaptor that enhances anterograde transport, where it activates and tethers kinesin-1 to the microtubule surface, enabling it to robustly transport mitochondria over long distances and through dense tau patches along microtubules (83). While several adaptors have been identified to control the motility of different organelles and vesicular cargoes, those that associate with phagosomes are not fully characterized. Yet, different adaptor–motor complexes likely associate with EPs and LPs and contribute to differences in their motility and responses to tau by regulating both the recruitment and activity of phagosome-bound motors.

### How are cargoes with different sets of motors selectively regulated along tau-decorated microtubules?

Emerging evidence also suggests that motor detachment and reattachment kinetics play a pivotal role during transport and could influence how cargoes respond to MAPs. Kinesin-1 provides more sustained force during transport but reattaches slowly after it detaches from the microtubule, whereas kinesin-2 and kinesin-3 frequently detach from microtubules under load but reattach much faster than kinesin-1 (84, 85). Because their binding rates are much faster than their unbinding rates, kinesin-2 and kinesin-3 can more efficiently probe for available landing sites along microtubules, which helps tether the cargo close to the microtubule and navigate around obstacles. These studies could explain how cooperation between motors, for instance those that produce force and others that may act as a tether, enable cargoes to endure long distance travel over several microns (Figs. 1*B* and 2, *A* and *B*). Although EPs travel farther distances, because they are bound by fewer types of motors, they may be more prone to inhibition by tau than cargoes that are bound by multiple types of motors like LPs, in which more kinesin and dynein motors engage the microtubule simultaneously to bypass tau (6). While our results suggest that altering the number and types of motors on a cargo as they mature changes the overall transport and response to tau, there are several other nonmutually exclusive mechanisms that regulate how motors engage with the microtubule surface as well as respond to MAPs. Thus, differences in membrane fluidity (68, 69), clustering of motors (46), as well as the type and number of regulatory adaptors present on the cargo surface are all possible factors that contribute to why the sets of dynein on EPs are more inhibited than those transporting LPs.

In neurons, several mechanisms must coordinate to ensure that cargoes are delivered with precision over long distances. MAPs that differentially impact the motility of kinesin and dynein motors compete for space along microtubules to govern the transport of intracellular cargoes (24, 26, 86). Microtubules within the axon that are decorated by tau could modulate directional transport or function as selective tracks for certain cargo, whereas microtubules that are bound by different MAPs act as selective tracks for other types of cargo. Our results suggest that tau functions as a barrier along microtubules to selectively inhibit some cargoes while allowing the passage of others depending on the type of motors driving their transport. Tau is also regulated by posttranslational modifications and isoform expression, which could influence its affinity for microtubules as well as its impact on the motility of different motors. Our studies indicate that regulating tau dynamics through posttranslational modifications or disease mutations would be expected to have cargo-specific effects on transport.

## Experimental procedures

### Cell culture and phagosome isolation

J774A.1 mouse macrophage (ATCC) were maintained in Dulbecco’s modified Eagle’s medium (Gibco), supplemented with 10% fetal bovine serum (Thermo Fisher Scientific) and 1% GlutaMAX (Gibco) at 37 °C with 5% CO_2_. Macrophages were plated in 4 10 cm cell culture dishes and grown to ∼80% confluency at 37 °C with 5% CO_2_. Phagosome isolation was performed as described previously (87). Beads were coated with 10% bovine serum albumin (BSA) and incubated in cells for 30 min or 90 min to isolate EPs or LPs, respectively. While 200 nm fluorescent beads were used for motility assays, larger 500 nm beads (yellow-green fluorescent; F8813, Invitrogen) were used for optical trapping and nonfluorescent beads were used for immunofluorescence imaging experiments. After treatment with beads, cells were washed with cold PBS and collected using a cell scrapper and spun at 650*g* at 4 °C for 5 min. Cells were resuspended in motility assay buffer (MAB; 10 mM Pipes, 50 mM K-acetate, 4 mM MgCl_2_, and 1 mM EGTA, pH 7.0.) supplemented with protease inhibitor cocktail (BioShop), 10 mM DTT, 1 mM MgATP, and 8.5% sucrose and transferred to a tight-fitting Dounce cell homogenizer and homogenized on ice by hand with 5 to 10 up-and-down strokes to ensure adequate disruption of cell membranes. Following, the cell homogenate was spun at 650*g*, and ∼1 ml of supernatant was mixed with 62% sucrose solution at a 1:1.2 ratio. All sucrose solutions were supplemented with protease inhibitor cocktail, 10 mM DTT, and 1 mM MgATP. The homogenate mixture was loaded on top of a 3.92 ml 62% sucrose cushion in an Ultra-Clear 5/8” X 4” Beckman Coulter tube (344061). The remaining sucrose solutions 2.61 ml 35% (or 30% for 200 nm beads), 2.61 ml 25%, and 2.61 ml 10% were added on top of the ∼2 ml homogenate mixture. The sucrose gradient was centrifuged in a swinging bucket rotor at 73,000*g* for 72 min at 4 °C. The bead-containing phagosomes appeared as a thin band at the 10 to 25% (or 25–30% for 200 nm beads) sucrose interface and extracted using a 21-gauge needle and syringe. Isolated phagosomes were kept on ice at 4 °C or flash frozen and stored at −80 °C.

### Polarity-marked microtubules

Bright double-cycled GMPCPP microtubule seeds were prepared as described in Hyman *et al.* (88), by mixing 25% Alexa647-labeled tubulin and 75% unlabeled tubulin in BRB80 (80 mM K-Pipes, 1 mM MgCl_2_, 1 mM EGTA, pH 6.8) to a final concentration of 5 mg/ml supplemented with 1 mM GTP (Sigma Aldrich) and polymerized at 37 °C for 20 min. Bright microtubule seeds were then vortexed in cold BRB80 supplemented with 1 mM GMPCPP (Jena Bioscience), 1 mM MgSO_4_, and 2 mM MgCl_2_ and incubated on ice for 5 min. This mixture was incubated at 37 °C for 30 min and pelleted at 30 psi for 5 min in the Beckman Airfuge using the A95 rotor. The pellet was resuspended in 70 μl cold BRB80 and depolymerized on ice for 20 min. The solution was mixed with 1 mM MgSO_4_, 2 mM MgCl_2_, and 1 mM GMPCPP and incubated on ice for 5 min. The mixture was polymerized at 37 °C for 30 min then spun at 30 psi for 5 min. The pellet was resuspended in BRB80 and aliquoted in 5 μl stocks, flash frozen, and stored at −80 °C. To prepare polarity-marked microtubules, GMPCPP seeds were warmed at 37 °C for 1 min and added to a mixture of 8% labeled tubulin and 92% unlabeled tubulin to a final concentration of 2 mg/ml in BRB80 and 1 mM GTP and incubated for 25 min at 37 °C. Microtubules were then stabilized with 20 μM Taxol (Cytoskeleton) and incubated for 25 min at 37 °C. Microtubules were cleared 2X by pelleting them at 10,600*g* for 5 min at RT then washed with T-BRB80 (BRB80 supplemented with 20 μM Taxol).

### Live cell imaging

Macrophages were plated in MatTek 35 mm glass-bottom dishes (no. 1.5) and grown to ∼50% confluency at 37 °C with 5% CO_2_. To perform live imaging, cells were treated with 200 nm blue fluorescent latex beads (F8805, Invitrogen) coated with 10% BSA diluted 1:100 in Dulbecco’s modified Eagle’s medium media and incubated at 37 °C with 5% CO_2_. To image EPs, cells were incubated for 30 min with beads prior to imaging, and to image LPs, cells were incubated for 90 min. Following, cells were washed 1X with PBS to remove nonphagocytosed beads and media was replenished with phenol red-free Leibovitz L-15 Media (Gibco). Live cell imaging was performed on an Eclipse Ti-E inverted microscope (Nikon) with custom optics for TIRF and imaged using EMCCD camera (iXon U897; Andor Technology) maintained at 37 °C. Time lapse recordings were acquired with 30 ms exposures using a 450 nm laser (100 mW) set at 1% power for 120 s per cell using Nikon Imaging Software-Elements acquisition software (Nikon). Image files were exported as TIFFs, which were opened with ImageJ (NIH) and bead containing phagosomes were tracked using TrackMate (47). Phagosomes were first identified using a Laplacian of Gaussian filter to detect the fluorescence signals. Detected spots were then tracked with the Linear Assignment Problem tracker and linked from frame-to-frame to trace trajectories of EPs and LPs. The tracking parameters were set to link tracks with distances < 0.5 μm and with gaps of two frames if the cargoes were temporarily out of frame. The *xy*-coordinates of each trajectory were imported into MATLAB (The MathWorks) to analyze the mode of transport, directionality, displacement, and number of reversals for all trajectories within cells using custom scripts.

To determine microtubule orientation, cells were treated with SiR-tubulin as per manufacturer’s instructions (Cytoskeleton) and imaged using TIRF after 4 h with 500 ms exposure and 1% laser power. Microtubules were monitored and the direction of polymerization was scored as plus-ends pointing inwards (towards the nucleus), outwards (towards the periphery), or tangentially to the center of the cell.

### Immunofluorescence imaging

Isolated EPs and LPs were added to silanized coverslips mounted to microscope slides using vacuum grease and double-sided tape to make flow chambers (89) and incubated for 1 h at RT to allow them to adhere to the surface. Following the incubation, chambers were washed 2X with MAB before and after treatment with Pluronic F-127 to prevent nonspecific binding before incubation with primary antibodies. Phagosomes were incubated with different combinations of primary antibodies for kinesin-1 (MAB1614, EMD Millipore), kinesin-2 (ab24626, Abcam) conjugated to Alexa568 (A20184, Thermo Fisher Scientific), dynein (sc-9115, Santa Cruz), kif16b (SAB1401759, Sigma), and kif1b (MABC309, EMD Millipore) for 1 h at RT in the dark. Following treatment with primary antibodies, phagosomes were washed 3X with MAB and incubated with secondary antibodies against mouse (Alexa488; A11029, Thermo Fisher Scientific) and rabbit (Alexa647; A32733, Thermo Fisher Scientific) for 1 h at RT in the dark. Samples were washed 3X with MAB before imaging. Multichannel images were acquired in brightfield or with 500 ms exposures in TIRF using the 488 nm, 561 nm, and 640 nm lasers, set between 1 and 5% power. Thresholds >800 a.u. were applied to compare the type of motors on EPs and LPs. Only phagosomes with fluorescence signals above the threshold were counted.

### Stepwise photobleaching

Phagosomes were incubated in flow chambers for 1 h and treated with Pluronic F-127 as described above. Separate chambers were used for each motor. Phagosomes were incubated with one of the following mouse monoclonal antibodies against kinesin-1 (MAB1614, EMD Millipore), kinesin-2 (ab24626, Abcam), kif16b (SAB1401759, Sigma), kif1b (MABC309, EMD Millipore), and dynein (MAB1618, EMD Millipore) for 2 h at RT in the dark. Chambers were washed 3X with MAB and incubated with anti-mouse Alexa647 (A21236, Thermo Fisher Scientific) secondary antibody for 1.5 h. Following, chambers were washed 3X with MAB. Phagosomes were imaged in epifluorescence with 500 ms exposures using a 640 nm laser at 3 mW. Recombinant rat kinesin-1 (rkin430-GFP (kif5c); A gift from Dr Gary Brouhard, Dept. Biology, McGill University) and mouse kinesin-2 (kif3a/b; A gift from Dr Susan Gilbert, Rensselaer Polytechnic Institute), and human kinesin-3 (kif16b-mCit; A gift from Dr Kristen J. Verhey, University of Michigan) overexpressed in Cos7 cells and extracted as described (90) were imaged under similar conditions to determine the number of steps for a single motor. Dilute single motors were incubated with primary and secondary antibodies and bound to unlabeled microtubules using 1 mM AMPPNP to detect single statically bound molecules. Images were acquired similar to phagosomes but instead using TIRF to achieve detectable steps. Only steps > 15 a.u. were considered. A step-finding algorithm (52) was used to determine the number and size of photobleaching steps for single motors and for the sets of motors on phagosomes. The mean step size was determined to be 21 a.u. for kinesin-1, kinesin-2, kif16b, and dynein, and 66 a.u. for recombinant single kinesin motors. The number of steps for each motor was determined by dividing the difference between the initial and final fluorescence intensity by the mean step size. Kinesins-1, -2, and -3 photobleached with a mean of ∼4 steps (Fig. S2*C*) and previous work from Chaudhary (91) showed that single dynein motors and kinesin-1 had similar size and number of photobleaching steps. The number of motors on EPs and LPs were estimated by dividing the number of steps of each motor on cargoes by the mean number of steps for single motors.

### *In vitro* motility assays

Flow chambers were first incubated with anti-β-tubulin (T4026 clone TUB2.1, Sigma) diluted 1:25 in BRB80 for 5 min. Chambers were then treated with F-127 for 5 min and washed 2X with T-BRB80. Polarity-marked microtubules were added to the chambers and incubated for 5 min at RT. Unbound microtubules were washed out with 2X T-BRB80. Isolated phagosomes were flown through the chamber supplemented with 0.2 mg/ml BSA, 10 mM DTT, 1 mM MgATP, 20 μM Taxol, 15 mg/ml glucose, ≥2000 units/g glucose oxidase, ≥6 units/g catalase, and 1 mg/ml casein. For experiments with tau, microtubules were incubated with 10 nM of Alexa568-labeled 3RS-tau for 30 min. Tau protein was expressed and purified as previously described in Chaudhary *et al.* (6). Phagosome mixtures were also supplemented with 10 nM tau. Using TIRF, microtubules and tau were imaged for one frame with the 561 nm and 640 nm lasers at 1 to 10% power, and time lapse recordings of phagosomes were imaged with 80 ms exposures using a 450 nm laser set at 2% power for 5 min.

### Subpixel tracking and change point analysis

Motility events were analyzed using Fluorescence Image Evaluation Software for Tracking and Analysis (FIESTA) (56). Phagosome fluorescence signals were fitted using the symmetrical 2D-Gaussian function and trajectories analyzed using custom MATLAB scripts. Change point analysis was applied to identified periods of stationary, diffusive, or processive periods of motility for each trajectory by finding the local α-values using a rolling MSD analysis over a sliding window length of 12 frames. Change points occurred when the difference between two adjacent α-values was >0.3 with a minimum of three frames between them. The tracking uncertainty was determined to be 16 nm from the tracked positions of nonmotile phagosomes (Fig. S4*A*). Runs were identified as stationary (R_L_ ≤ 16 nm), diffusive (R_L_ > 16 nm and α ≤ 1), or processive (R_L_ > 16 nm and α > 1). Runs were further categorized as plus-end–directed or minus-end–directed and analyzed to determine the velocities, run lengths, and time intervals for each mode of transport. MSD analysis was used to determine the minimum run length of processive runs based on the threshold used for change point analysis (Fig. S4*B*). Processive runs with run lengths ≥150 nm were further analyzed since tau did not have any apparent effect on stationary, diffusive, or processive runs <150 nm.

### Optical trapping

Optical trapping was performed similar to motility assays, except using a 10 W, 1064 nm laser (IPG Photonics) to trap phagosomes, a quadrant photodiode detector (PDP90A, Thorlabs) to sense the displacement, and forces exerted by phagosomes away from the trap center and TIRF microscopy to position phagosomes over microtubules. The trap stiffness and positional calibration was determined by fitting a Lorentzian function to the power spectrum of thermal fluctuations of trapped phagosomes. Forces were calculated by measuring the displacement of the trapped phagosome from the trap center, where the displacement is directly proportional to the trap stiffness. Forces were filtered by stall time and analyzed using custom MATLAB codes to measure the magnitudes, frequency of events, and the relative binding and unbinding rates (24, 66).

### Quantification of the number of motors on cargo

To estimate the number and ratio of motors on individual cargo, we first calculated the fraction of beads that were enveloped in a phagosome ($F_{\;{\{Ph}_{+}\}}$) from the multicolor immunofluorescence imaging analysis (Fig. 1*E*). For each image, beads ($B_{\left\{TOTAL\right\}}$) that were isolated and adequately distanced from neighboring beads so to ensure that fluorescence signals did not overlap were counted. Beads that were immunolabeled for dynein, kinesin-1, kinesin-2, or any combination of these motors were considered phagosomes ($B_{\left\{+\right\}}$) and those that were not labeled considered nonphagocytosed beads. The fraction of beads that were phagosomes was determined as follows:

$$F_{\left\{{Ph}_{+}\right\}\;}=\;\frac{B_{\left\{+\right\}}}{B_{\left\{TOTAL\right\}}}$$

Since the ratio of phagocytosed beads to nonphagocytosed beads isolated from EPs and LPs were different, we corrected the fraction of beads that were immunolabeled, where M is either kinesin-1, kinesin-2, kinesin-3, or dynein, and ${Ph}_{+M}$ is the corrected fraction of phagosomes with a positive signal for M motor (Fig. S1*I*). Bootstrapping was used to determine the mean and 95% confidence intervals of the total number of phagosomes bound by each motor M.

$${Ph}_{\;+\;M}\;=\;\frac{B_{\left\{+M\right\}}}{\frac{B_{\left\{TOTAL\right\}}}{F_{\left\{{Ph}_{+}\right\}\;}}}$$

Next, the mean number of each motor on individual phagosomes was determined. First, the total number of phagosomes that were bound by one or more motors of a given type M was calculated as follows:

$${Ph}_{\left\{\geq 1M\right\}}={Ph}_{\left\{1M\right\}}\;+\;{Ph}_{\left\{2M\right\}}\;+\;\ldots \;+\;{Ph}_{\left\{nM\right\}}$$

Where ${Ph}_{\left\{1M\right\}}$ is the number of phagosomes that contain 1 M motor, ${Ph}_{\left\{2M\right\}}$ is the number of phagosomes that contain 2 M motors, ${Ph}_{\left\{nM\right\}}$ is the number of phagosomes that contain *n* number of M motors, and ${Ph}_{\left\{\geq 1M\right\}}$ is the total number of phagosomes that contain one or more number of M motors.

Next, we calculated the number of phagosomes that were not bound by a given motor M as follows:

$${Ph}_{\left\{0M\right\}}={Ph}_{\left\{\geq 1M\right\}}\times \left(\frac{1-\;{Ph}_{+M}}{{Ph}_{+M}}\right)$$

Where, ${Ph}_{\left\{0M\right\}}$ is the number of phagosomes that were not bound by a given motor M but bound by other types of motors ≠M . Thus, the total number of phagosomes bound by 0 to n number of each type of M motor was calculated as follows:

$${Ph}_{\left\{M_{TOTAL}\right\}}={Ph}_{\left\{0M\right\}}+{Ph}_{\left\{1M\right\}}+{Ph}_{\left\{2M\right\}}+\ldots \;+\;{Ph}_{\left\{nM\right\}}$$

Next, we determined the total number of each motor ($M_{TOTAL}$) among the total number of phagosomes (${Ph}_{\left\{M_{TOTAL}\right\}}$), then calculated the mean number of motors ($\mu_{\left\{M_{TOTAL}\right\}}$) on individual phagosomes using bootstrapping as follows (Fig. 1*H*):

$$M_{TOTAL}=\left(1\times {Ph}_{\left\{1M\right\}}\right)+\left(2\times {Ph}_{\left\{2M\right\}}\right)+\ldots \;+\;\left(n\times {Ph}_{\left\{nM\right\}}\right)$$

$$\mu_{\left\{M_{TOTAL}\right\}}=\frac{M_{TOTAL}}{{Ph}_{\left\{M_{TOTAL}\right\}}}$$

### Simulations of motor sets and estimation of the total number of motors on each cargo

To estimate the total number of motors on individual EPs and LPs, we generated populations of cargoes with simulated motor sets from the experimental data. First, the cumulative distribution function (CDF) was constructed from the distribution of phagosomes bound by 0 to n number of M motors (Fig. S2*G*). Then, 1000 uniform random numbers were generated from the empirical distributions and interpolated from the CDF of each motor M. Thus, simulated populations containing 1000 cargoes were generated with the same probabilities of cargo bound by 0 to n number of M motors as observed experimentally.

To determine the total number of motors on individual phagosomes, the following equation was used:

$$m=\left(m_{kin1}+m_{kin2}+m_{kin3}+m_{dyn}\right)$$

Where m is the sum of the number of kinesin-1 ($m_{kin1}$), kinesin-2 ($m_{kin2})$, kinesin-3 ($m_{kin2})$, and dynein ($m_{dyn}$) on a single cargo. The mean number of motors on individual cargoes within the simulated populations was calculated using bootstrapping as follows:

$$\mu_{\left\{m\right\}}=\;\frac{1}{N}\;\sum_{i=1}^{N}m_{i}$$

Where N is the population size and $\mu_{\left\{m\right\}}$ is the mean total number of motors on individual cargoes within the population.

### Modeling cargo transport by different sets of motors

Mathematical modeling based on stochastic tug-of-war used to describe bidirectional transport was expanded to include transport by kinesins-1, -2, -3, and dynein-dynactin-BicD2 (Müller *et al.*, 2008). Motility and force parameters from single molecule studies were applied to the model to describe the collective transport of cargoes by teams of kinesins-1, -2, -3 and dynein-dynactin-BicD2 (Table S1). To generate small changes in the number of motors bound to cargoes, 100 uniformly random numbers between the 40th and 60th quantiles were generated from the empirical distributions and interpolated from the CDF of each motor M (Fig. S2*G*). The change in run lengths and directionality were compared to show that modeling the transport of populations of cargoes with small variations in the sets of motors predicts the transport of cargoes observed *in vitro* and in cells.

### Statistical analysis

All data were presented with error bars indicating SEM or 95% confidence intervals when specified in the figure legends. All *n* and number of replicates were mentioned in the figure legends. Bootstrapping analysis was performed in MATLAB and used to test for statistical significance and determine confidence intervals. Fisher’s exact test was used to determine the significance of the frequency of plus-end– and minus-end–directed EPs that paused, passed, or detached from the microtubule when they encountered tau. Kolmogrov–Smirnov test were used to test for statistical significance of tau’s effect on the distribution of run lengths, velocities, force events, and relative binding rates. The binomial parameter estimates test (MATLAB) was used to determine the confidence intervals of the binominal proportion of bidirectional force events (Fig. 5).

## Data availability

All data are contained within the manuscript.

## Supporting information

This article contains supporting information (6, 33, 52, 54, 55, 59, 61, 63, 65, 84, 85, 92, 93, 94, 95, 96, 97).

## Conflict of interest

The authors declare that they have no conflicts of interest with the contents of this article.
