# Supplementary material for: The types and numbers of kinesins and dyneins transporting endocytic cargoes modulate their motility and response to tau
Source: J Biol Chem. 2024 Apr 25;300(6):107323. doi: 10.1016/j.jbc.2024.107323 (PMC11130734; doi:10.1016/j.jbc.2024.107323)
Supplement: Supporting Information [file mmc1.pdf]

The type and numbers of kinesins and dyneins transporting endocytic cargoes modulate their motility and response to tau

Daniel Beaudet, Christopher L. Berger, Adam G. Hendricks

Supporting information

This PDF file includes:

Supplemental Figures S1 to S6
Supplemental Table S1

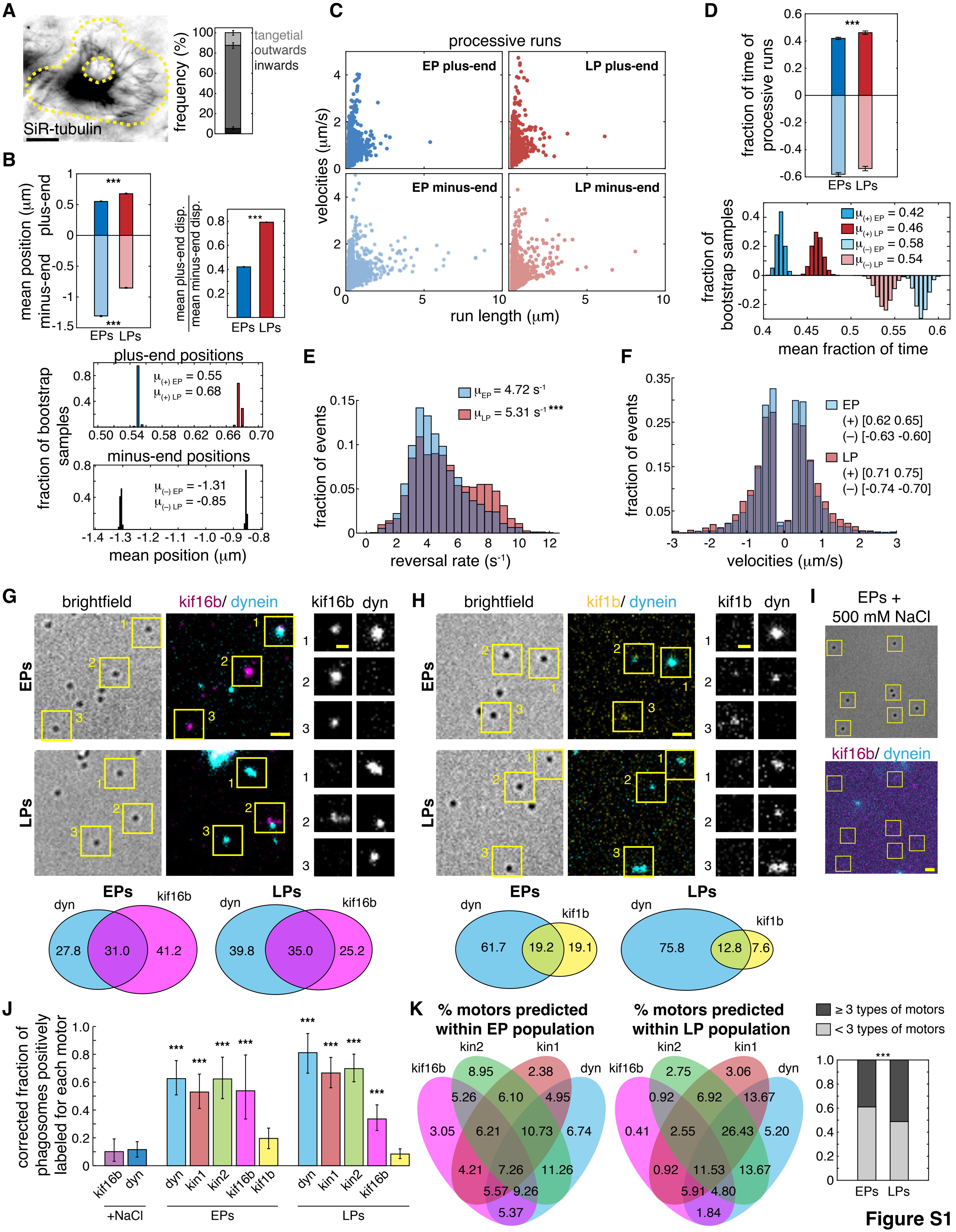

A
SiR-tubulin
frequency (%)
tangetial
outwards
inwards
B
mean position (μm)
plus-end
minus-end
EPs LPs
mean plus-end disp. / mean minus-end disp.
plus-end positions
$\mu_{(+)EP}$ = 0.55
$\mu_{(+)LP}$ = 0.68
minus-end positions
$\mu_{(-)EP}$ = -1.31
$\mu_{(-)LP}$ = -0.85
fraction of bootstrap samples
mean position (μm)
C
processive runs
EP plus-end
LP plus-end
EP minus-end
LP minus-end
velocities (μm/s)
run length (μm)
D
fraction of time of processive runs
$\mu_{(+)EP}$ = 0.42
$\mu_{(+)LP}$ = 0.46
$\mu_{(-)EP}$ = 0.58
$\mu_{(-)LP}$ = 0.54
fraction of bootstrap samples
mean fraction of time
E
μ_{EP} = 4.72 s^{-1}
μ_{LP} = 5.31 s^{-1} ***
fraction of events
reversal rate (s^{-1})
F
EP
(+) [0.62 0.65]
(–) [-0.63 -0.60]
LP
(+) [0.71 0.75]
(–) [-0.74 -0.70]
fraction of events
velocities (μm/s)
G
brightfield
kif16b/ dynein
kif16b dyn
EPs
LPs
dyn kif16b
27.8 31.0 41.2
39.8 35.0 25.2
H
brightfield
kif1b/ dynein
kif1b dyn
EPs
LPs
dyn kif1b
61.7 19.2 19.1
75.8 12.8 7.6
I
EPs + 500 mM NaCl
kif16b/ dynein
J
corrected fraction of phagosomes positively labeled for each motor
kif16b dyn
dyn kin1 kin2 kif16b kif1b
dyn kin1 kin2 kif16b
+NaCl
EPs
LPs
K
% motors predicted within EP population
kin2 kin1 kif16b dyn
8.95 2.38 5.26 6.10 4.95 3.05 6.21 10.73 6.74 4.21 7.26 11.26 5.57 9.26 5.37
% motors predicted within LP population
2.75 3.06 0.92 6.92 13.67 0.41 2.55 26.43 5.20 0.92 11.53 13.67 5.91 4.80 1.84
≥ 3 types of motors
< 3 types of motors
EPs LPs

Figure S1

Figure S1. Related to Figure 1.

A) Image shows the maximum projection of J774A.1 mouse macrophage cell treated with SiR-tubulin. Cell periphery and nucleus are outlined in yellow. Scale bar is 10μm. The plot shows the frequency of microtubule plus-ends pointed inwards, outwards, or tangentially to the center of the cell (n = 9 cells). **B)** A bar graph shows the mean plus-end and minus-end positions of EP and LP trajectories in cells. To the right, a bar graph shows the ratio of the mean plus-end to mean minus-end displacements for EPs and LPs. Below, bootstrapping was performed to resample the data sets 10,000 times to determine the means (μ), 95% confidence intervals (CI), and test the statistical significance between mean positions. **C)** Plots show the velocities vs. run lengths of plus-end and minus-end directed processive runs ≥ 150 nm for EPs (n = 3652 plus-end runs; n = 4408 minus-end runs) and LPs (n = 2655 plus-end runs; n = 2768 minus-end runs) in cells. **D)** A bar graph shows the fraction of time of plus-end and minus-end directed processive runs for EPs and LPs. Bootstrapping was used to test the difference in the means and determine the error bars that show 95% CI. **E)** A bar graph shows the reversal rates of EPs and LPs. Bootstrapping was used to determine the statistical significance between the mean reversal rates of EPs and LPs. **F)** A plot shows the velocities of processive runs of EPs and LPs in the plus-end and minus-end directions. The numbers of runs are the same as in panel B. 95% CI are shown in the legend. **G and H)** Images show isolated EPs and LPs immunolabeled for G) kif16b and dynein and H) kif1b and dynein. To the right, zoomed in ROIs show the different combinations of motors on individual phagosomes. Below, Venn diagrams show the mean percentages of individual motors and combinations of G) kif16b and dynein on EPs (n = 83) and LPs (n = 109) and H) kif1b and dynein on EPs (n = 103) and LPs (n = 113) from 3 independent phagosome isolations. Scale bars are 2 μm or 1 μm for selected ROIs. **I)** Images show phagosomes treated with 500 mM NaCl to strip proteins from the outer membranes and immunolabeled for kif16b and dynein. Salt-stripped phagosomes were used as a non-specific labeling control for immunofluorescence experiments. Scale bar is 2 μm. **J)** A plot shows the corrected fraction of phagosomes immunolabeled for each motor (see methods). Bootstrapping was used to determine statistical significance and error bars that show 95% CI. **K)** Venn diagrams show the predicted percentages of cargo bound by kinesin -1, -2, -3, dynein, or various combinations of different motors on EPs (n = 969) and LPs (n = 980). Estimates are based on random sampling of the data obtained from multi-color immunofluorescence experiments (see methods). To the right, a bar graph shows the fraction of EPs and LPs with ≥ 3 different types of motors or < 3 different types of motors. (*** $p < 0.0001$).

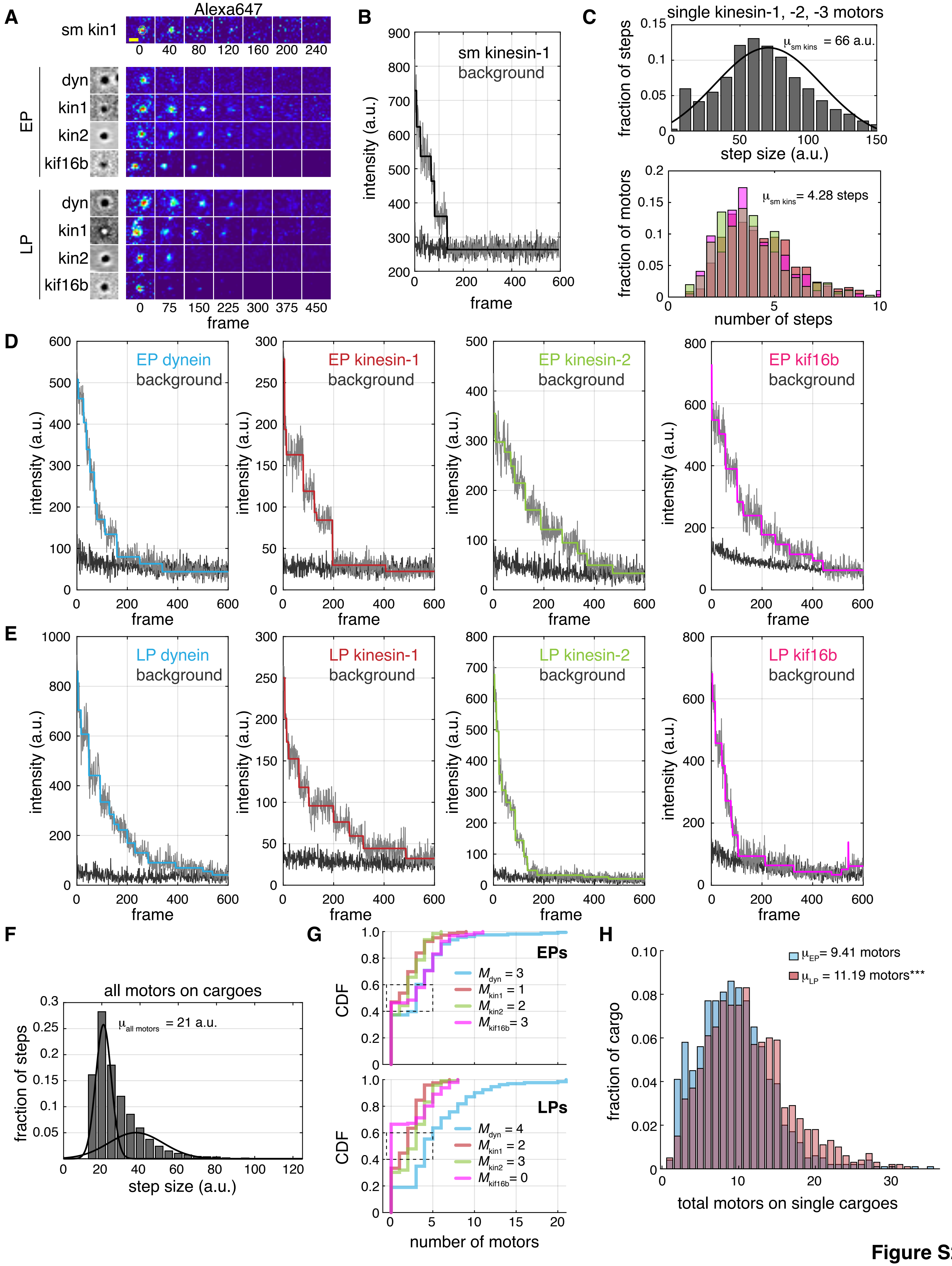
A
Alexa647
sm kin1
0 40 80 120 160 200 240
EP
dyn
kin1
kin2
kif16b
LP
dyn
kin1
kin2
kif16b
0 75 150 225 300 375 450
frame
B
sm kinesin-1
background
intensity (a.u.)
frame
C
single kinesin-1, -2, -3 motors
$\mu_{sm\ kins}$ = 66 a.u.
fraction of steps
step size (a.u.)
$\mu_{sm\ kins}$= 4.28 steps
fraction of motors
number of steps
D
EP dynein
EP kinesin-1
EP kinesin-2
EP kif16b
background
intensity (a.u.)
frame
E
LP dynein
LP kinesin-1
LP kinesin-2
LP kif16b
background
intensity (a.u.)
frame
F
all motors on cargoes
$\mu_{all\ motors}$ = 21 a.u.
fraction of steps
step size (a.u.)
G
EPs
M_{dyn} = 3
M_{kin1} = 1
M_{kin2} = 2
M_{kif16b}= 3
LPs
M_{dyn} = 4
M_{kin1} = 2
M_{kin2} = 3
M_{kif16b}= 0
CDF
number of motors
H
μ_{EP}= 9.41 motors
μ_{LP} = 11.19 motors***
fraction of cargo
total motors on single cargoes

Figure S2

Figure S2. Related to Figure 1.

A) Time lapse images of photobleaching experiments show how the fluorescence intensity of a single kinesin-1 motor (sm kin1), or multiple dynein (dyn), kinesin-1 (kin1), kinesin-2 (kin2), or kif16b motors on EPs and LPs decrease over time. Scale bar is 1 μm. **B)** The trace shows the stepwise decrease in fluorescence signal of a single motor of kinesin-1. **C)** A plot shows the distribution of the step sizes for all single kinesin-1 ($n = 472$), kinesin-2 ($n = 134$), and kif16b ($n = 195$) motors from 2 independent experiments. The mean unitary step size was determined to be 66 a.u. Single motors were imaged in TIRF. **D** and **E)** Traces show the stepwise decrease in fluorescence signal compared to background for dynein, kinesin-1, kinesin-2, and kif16b on D) EPs and E) LPs. A step-finding algorithm based on the Student's t-test (16, 17) was used to determine the number and the size of steps for each trace. **F)** A plot shows the distribution of step sizes for kinesin-1, kinesin-2, kif16b, and dynein motors counted on EPs and LPs. A Gaussian Mixture Model was used to determine the mean step size for a single fluorophore. The mean step size was determined to be 21 a.u., which is different from the mean step size of a single motor because phagosomes were imaged using epifluorescence microscopy. **G)** Cumulative distribution function (CDF) plots show the fraction of phagosomes bound by 0–22 kinesin-1, kinesin-2, kif16b, and dynein motors. Median (*M*) number of motors are indicated in the legends. Dashed boxes show the distribution of the number of motors on cargoes within the 40th and 60th quantiles. **H)** A histogram shows the distribution of the predicted total number of motors on individual EPs and LPs ($n = 1000$ each; see methods). Bootstrapping was used to calculate the means and determine the statistical significance (*** $p < 0.0001$).

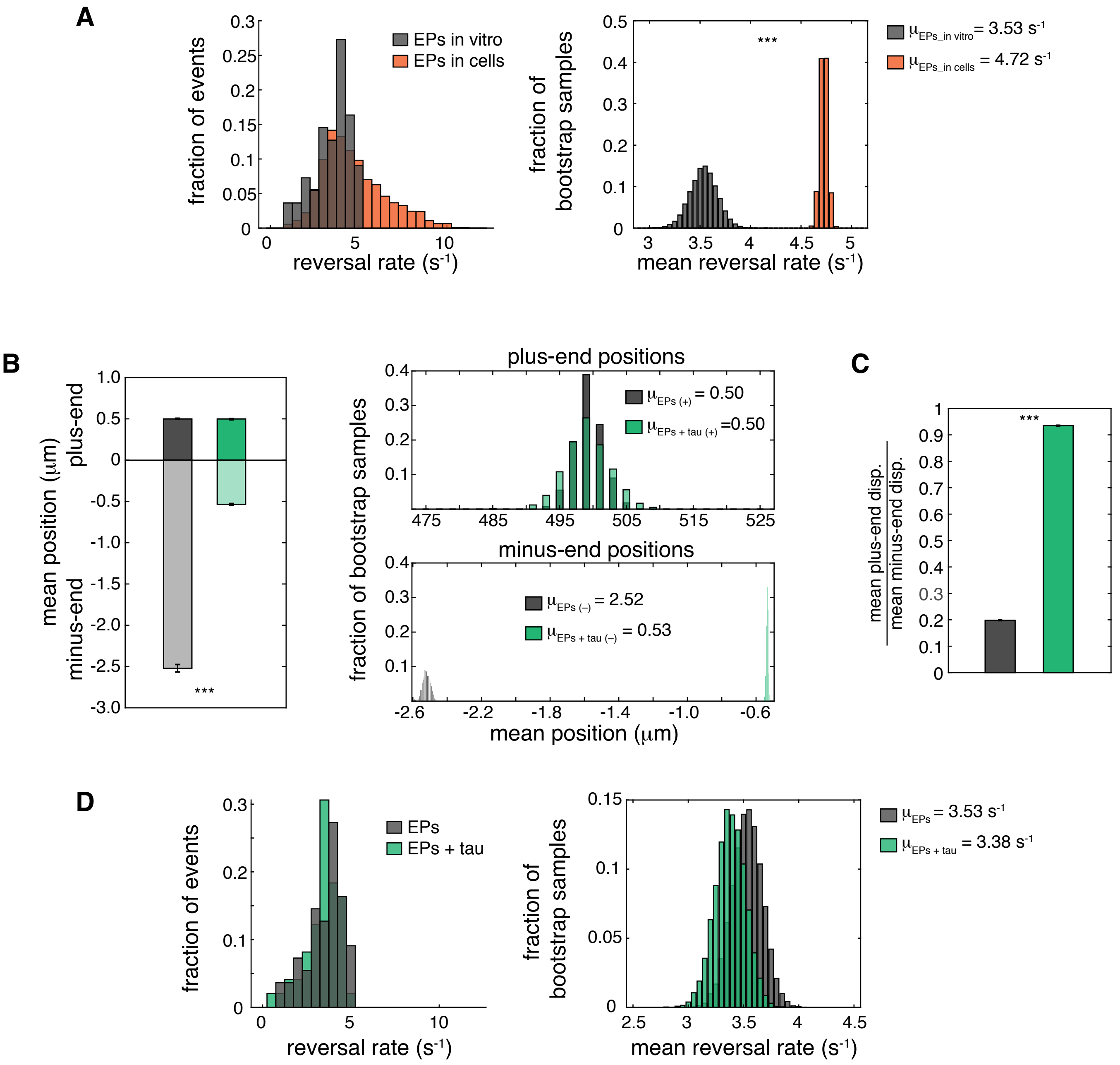
A
fraction of events
EPs in vitro
EPs in cells
reversal rate (s-1)
fraction of bootstrap samples
***
mean reversal rate (s-1)
μEPs_in vitro = 3.53 s-1
μEPs_in cells = 4.72 s-1
B
mean position (μm)
plus-end
minus-end
***
plus-end positions
μEPs (+) = 0.50
μEPs + tau (+) =0.50
minus-end positions
μEPs (–) = 2.52
μEPs + tau (–) = 0.53
fraction of bootstrap samples
mean position (μm)
C
***
mean plus-end disp.
mean minus-end disp.
D
fraction of events
EPs
EPs + tau
reversal rate (s-1)
fraction of bootstrap samples
mean reversal rate (s-1)
μEPs = 3.53 s-1
μEPs + tau = 3.38 s-1

Figure S3

Figure S3. Related to Figure 2.
A) A bar graph compares the reversal rates of EPs in cells and in vitro. Bootstrapping was used to determine the statistical significance between the mean reversal rates of EPs in cells and in vitro. **B)** A bar graph shows the mean plus-end and minus-end positions of EPs +/- tau in vitro. Bootstrapping was used to determine the means (μ), 95% CI shown by the error bars, and test the statistical significance of the mean positions. **C)** A bar graph shows the ratio of plus-end to minus-end mean displacements for EPs +/- tau. **D)** A bar graph shows the reversal rates of EPs +/- tau. To the right, bootstrapping was used to determine the statistical significance between the mean reversal rates of EPs +/- tau (not significant). (*** $p < 0.0001$).

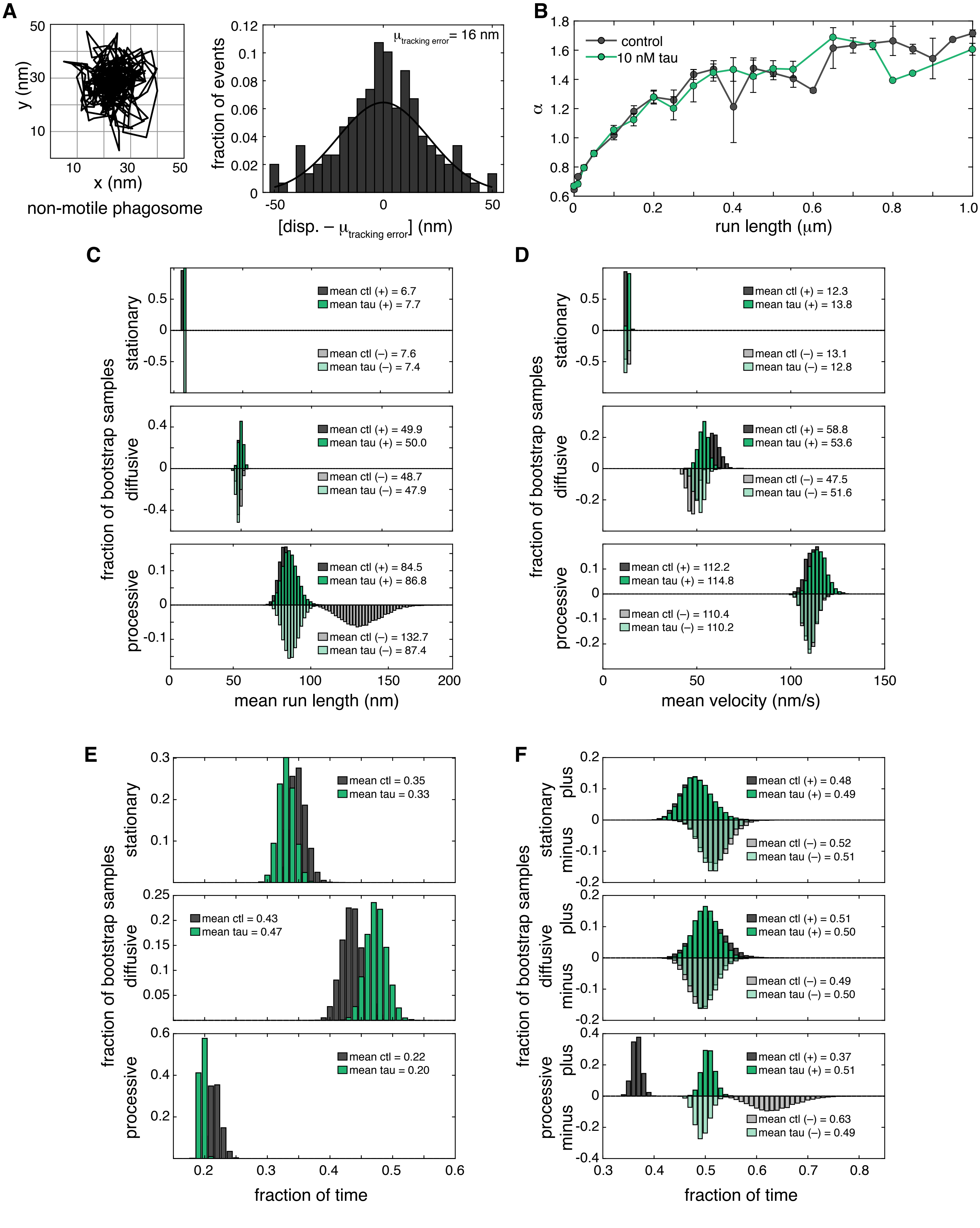
A
50
30
10
y (nm)
x (nm)
non-motile phagosome
fraction of events
$\mu_{tracking\ error}$ = 16 nm
[disp. – $\mu_{tracking\ error}$] (nm)
B
control
10 nM tau
α
run length (μm)
C
stationary
diffusive
processive
fraction of bootstrap samples
mean ctl (+) = 6.7
mean tau (+) = 7.7
mean ctl (–) = 7.6
mean tau (–) = 7.4
mean ctl (+) = 49.9
mean tau (+) = 50.0
mean ctl (–) = 48.7
mean tau (–) = 47.9
mean ctl (+) = 84.5
mean tau (+) = 86.8
mean ctl (–) = 132.7
mean tau (–) = 87.4
mean run length (nm)
D
mean ctl (+) = 12.3
mean tau (+) = 13.8
mean ctl (–) = 13.1
mean tau (–) = 12.8
mean ctl (+) = 58.8
mean tau (+) = 53.6
mean ctl (–) = 47.5
mean tau (–) = 51.6
mean ctl (+) = 112.2
mean tau (+) = 114.8
mean ctl (–) = 110.4
mean tau (–) = 110.2
mean velocity (nm/s)
E
mean ctl = 0.35
mean tau = 0.33
mean ctl = 0.43
mean tau = 0.47
mean ctl = 0.22
mean tau = 0.20
fraction of time
F
plus
minus
mean ctl (+) = 0.48
mean tau (+) = 0.49
mean ctl (–) = 0.52
mean tau (–) = 0.51
mean ctl (+) = 0.51
mean tau (+) = 0.50
mean ctl (–) = 0.49
mean tau (–) = 0.50
mean ctl (+) = 0.37
mean tau (+) = 0.51
mean ctl (–) = 0.63
mean tau (–) = 0.49
fraction of time

Figure S4

Figure S4. Related to Figure 3.

A) A plot shows the positional tracking of a non-motile phagosome used to determine the tracking error for motility assays. On the right, a histogram shows the displacement of non-motile phagosomes ($n = 9$) subtracted by the mean tracking error. **B)** A plot shows the MSD of runs parsed by run length for EPs +/- tau following change point analysis. The MSD was calculated to validate the change point analysis threshold used to identify diffusive and processive periods of motility. Typically, runs with lengths ≥ 150 nm were processive ($\alpha > 1$), while shorter runs were diffusive ($\alpha < 1$). Bootstrap analysis was performed to test the impact of tau on the average **C)** run length and **D)** velocity for stationary, diffusive and processive runs, **E)** the fraction of time of stationary, diffusive, and processive runs, and **F)** the fraction of time of plus-end and minus-end directed stationary, diffusive and processive motility.

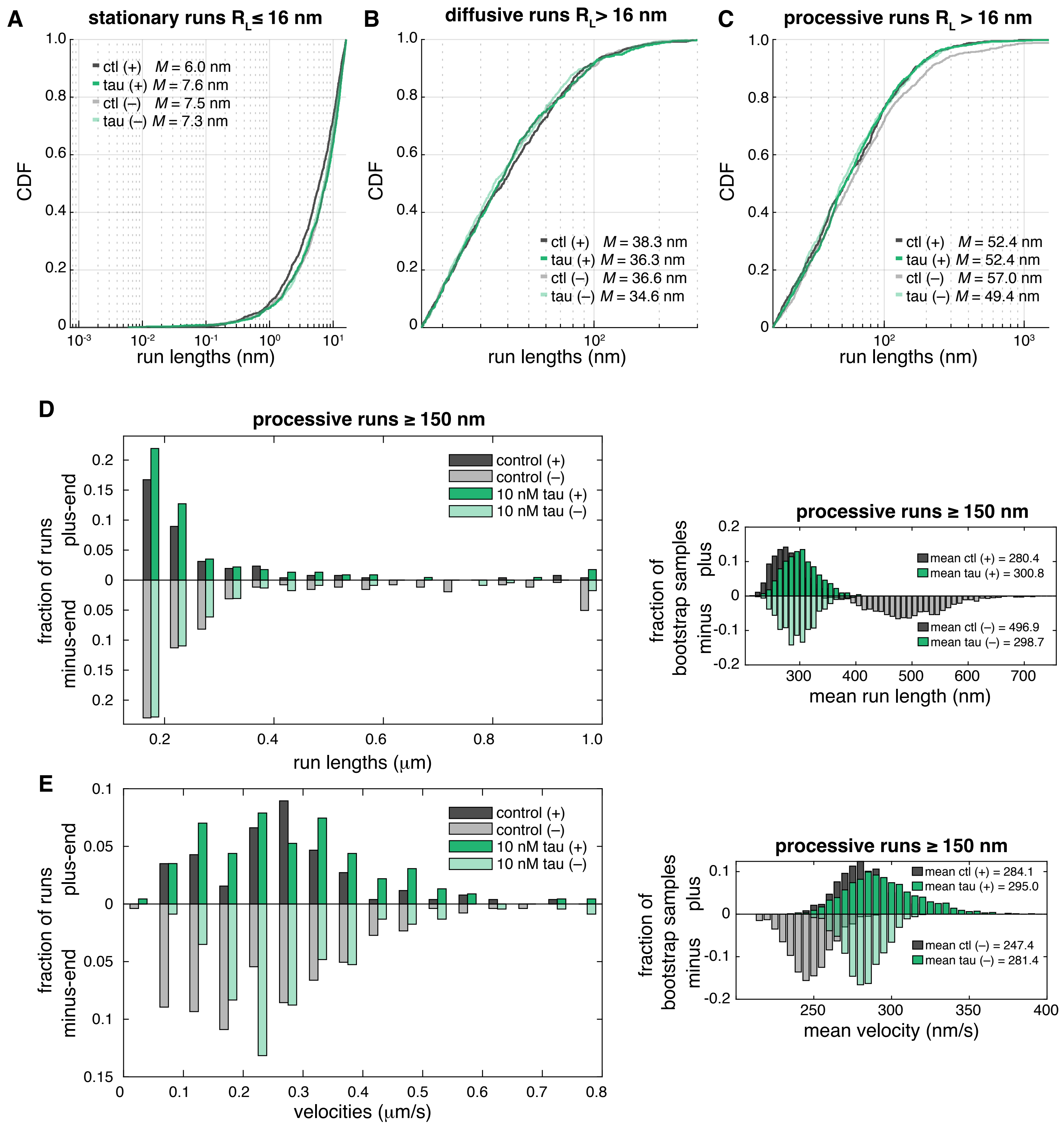

A
stationary runs $R_L \leq 16$ nm
ctl (+) $M = 6.0$ nm
tau (+) $M = 7.6$ nm
ctl (–) $M = 7.5$ nm
tau (–) $M = 7.3$ nm
CDF
run lengths (nm)
B
diffusive runs $R_L > 16$ nm
ctl (+) $M = 38.3$ nm
tau (+) $M = 36.3$ nm
ctl (–) $M = 36.6$ nm
tau (–) $M = 34.6$ nm
CDF
run lengths (nm)
C
processive runs $R_L > 16$ nm
ctl (+) $M = 52.4$ nm
tau (+) $M = 52.4$ nm
ctl (–) $M = 57.0$ nm
tau (–) $M = 49.4$ nm
CDF
run lengths (nm)
D
processive runs ≥ 150 nm
control (+)
control (–)
10 nM tau (+)
10 nM tau (–)
fraction of runs
plus-end
minus-end
run lengths (μm)
processive runs ≥ 150 nm
mean ctl (+) = 280.4
mean tau (+) = 300.8
mean ctl (–) = 496.9
mean tau (–) = 298.7
fraction of bootstrap samples
plus
minus
mean run length (nm)
E
control (+)
control (–)
10 nM tau (+)
10 nM tau (–)
fraction of runs
plus-end
minus-end
velocities (μm/s)
processive runs ≥ 150 nm
mean ctl (+) = 284.1
mean tau (+) = 295.0
mean ctl (–) = 247.4
mean tau (–) = 281.4
fraction of bootstrap samples
plus
minus
mean velocity (nm/s)

Figure S5

Figure S5. Related to Figure 4.
A–C) The CDF plots show how tau impacts the frequency of plus-end and minus-end directed A) stationary, B) diffusive, and C) processive run lengths for EPs +/- tau identified by change point analysis. Stationary runs were identified as any run with a run length (R_L) ≤ 16 nm, which was determined by calculating the tracking error (Fig S4A). Diffusive runs were categorized as runs with $\alpha < 1$ and $R_L > 16$nm and processive runs were identified as runs with $\alpha > 1$ and $R_L >$ 16nm. Tau reduced minus-end directed processive run lengths but did not significantly change stationary or diffusive run lengths (minus-end processive runs, $p < 0.05$ by two-sample Kolmogorov-Smirnov test). Median run lengths (*M*) are shown. Based on the MSD (Fig S4B), we analyzed the impact of tau on processive runs with lengths ≥ 150 nm. **D)** Histograms show the distribution of run lengths of plus-end and minus-end directed processive runs ≥ 150 nm +/- tau (95 plus-end and 162 minus-end control runs, and 112 plus-end and 116 minus-end runs with tau). **E)** The CDF of the velocities of plus-end and minus-end directed processive runs ≥ 150 nm +/- tau. Bootstrap analysis was performed to test tau's impact on the D) mean run lengths and E) mean velocities of plus-end and minus-end directed processive runs with $R_L \geq$ 150 nm.

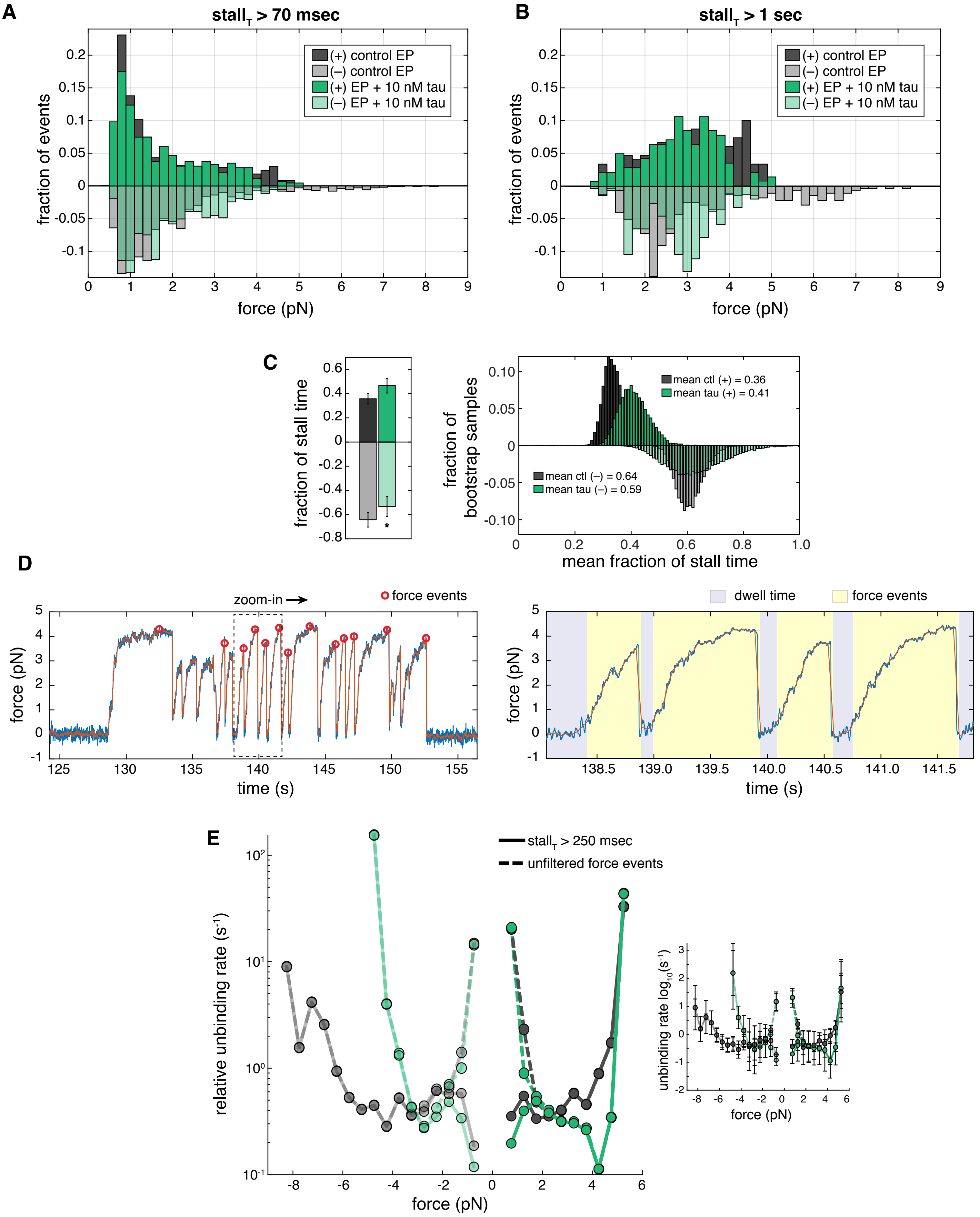
A
stall$_T$ > 70 msec
fraction of events
force (pN)
(+) control EP
(–) control EP
(+) EP + 10 nM tau
(–) EP + 10 nM tau
B
stall$_T$ > 1 sec
C
fraction of stall time
fraction of bootstrap samples
mean ctl (+) = 0.36
mean tau (+) = 0.41
mean ctl (–) = 0.64
mean tau (–) = 0.59
mean fraction of stall time
D
zoom-in
force events
dwell time
force (pN)
time (s)
E
stall$_T$ > 250 msec
unfiltered force events
relative unbinding rate (s^{-1})
unbinding rate log$_{10}$(s^{-1})
force (pN)

Figure S6

Figure S6. Related to Figure 5.
A and **B)** Histograms show the distribution of the total fraction of forces +/- tau in the plus-end and minus-end direction for A) events with stall times ($stall_T$) > 70 msec and B) longer sustained forces with $stall_T$ > 1 sec. Lower forces with shorter time intervals are common in both the plus-end and minus-end directions, which are thought to be due to the sets of motors detaching from the microtubule before maximum stall forces are reached. **C)** A bar graph shows the fraction of time of force events in the plus-end and minus-end direction +/- tau. Error bars indicate 95% CI. Bootstrapping analysis was used to test tau's impact on the fraction of plus-end and minus-end stall times. Means are shown (* $p < 0.05$). **D)** A plot shows an example force trace of a plus-end directed EP. On the left, red circles indicate force events with stall durations > 250 msec and stall forces > 0.5 pN. On the right, is a zoomed-in region from the left plot (dashed box) to show how the binding and unbinding rates were determined. The intervals of diffusive dwell times (purple) > 0.05 s are shown between force events (yellow). **E)** A plot shows the force-dependent relative unbinding rates calculated for plus-end and minus-end directed forces with stall durations > 250 msec (solid lines) and unfiltered forces (dashed lines) +/- tau. The plot to the right shows the log-unbinding rates on the y-axis. error bars indicate SEM.

EP modeling parameters

	- tau				+ tau			
	**kin1**	**kin2**	**kin3**	**DDB**	**kin1**	**kin2**	**kin3**	**DDB**
number of motors	0–2	0–2	0–3	1–3	0–2	2	0–3	1–3
k_{detach} (s^{-1})	0.96[1,2,3,4]	0.76[1,4]	0.10[6]	0.5[1,7,8,9]	**1.92**[13,14]	0.76	**0.20**[15]	**0.88**
k_{attach} (s^{-1})	1.9[1,4]	6.8[1,4]	18.6[1,2]	0.92[1,10]	1.9	6.8	18.6	0.92
F_{detach} (pN)	6.8[1,2,3]	3.0[3,5]	1.0[2,3]	∞[9]	6.8	3.0	1.0	∞
F_{stall} (pN)	6.0[1,2]	6.0[1,5]	6.0[1]	4.0[9,11,12]	6.0	6.0	6.0	4.0
Vel_{fwd} (nm/s)	586[1]	307[1]	950[6]	-360[9,11,12]	586	307	950	-360
Vel_{bwd} (nm/s)	-24	-24	-24	40	-24	-24	-24	40

LP modeling parameters

	- tau				+ tau			
	**kin1**	**kin2**	**kin3**	**DDB**	**kin1**	**kin2**	**kin3**	**DDB**
number of motors	1–2	2–3	0	4–5	1–2	2–3	0	4–5
k_{detach} (s^{-1})	0.96	0.76	0.10	0.5	**1.92**	0.76	**0.20**	**0.56**
k_{attach} (s^{-1})	1.9	6.8	18.6	0.92	1.9	6.8	18.6	0.92
F_{detach} (pN)	6.8	3.0	1.0	∞	6.8	3.0	1.0	∞
F_{stall} (pN)	6.0	6.0	6.0	4.0	6.0	6.0	6.0	4.0
Vel_{fwd} (nm/s)	586	307	950	-360	586	307	950	-360
Vel_{bwd} (nm/s)	-24	-24	-24	40	-24	-24	-24	40

Table S1

Table S1 related to Figure 6.
Motility and force parameters from single molecule studies were used to describe the collective transport of cargoes by teams of kinesins -1, -2, -3, and dynein-dynactin-BicD2 (DDB). The number of motors on EPs and LPs varied to mimic the heterogeneity in the sets of motors found experimentally (Fig S2G). The attachment rates (k_{attach}) obtained from Gicking et al., (1) were reduced to account for differences between the in vitro and cellular environments. In the presence of tau, the unloaded unbinding rates (k_{detach}) of kinesin-1, kinesin-3, and DDB for EPs and LPs increased. Based on results from this study and Chaudhary et al., (16), we modeled the effects of tau on DDB to be ~ twice as inhibitory on EPs than LPs. Retrograde transport was modeled using single molecule parameters of DDB, which is more processive and exerts higher forces than dynein alone, resembling the active dynein-dynactin-adaptor complexes found on endogenous cargo (9, 11, 12). DDB was modeled as forming an ideal bond with a microtubule under hindering load, where the detachment force (F_{detach}) of DDB was >> than that of any kinesin motor (1). Kif1a single-molecule motor parameters were mostly used to describe the motility of kinesin-3 since there are limited data available that describe the motility parameters of kif16b (1).

Supplemental references

1. Gicking, A.M., Ma, T.C., Feng, Q., Jiang, R., Badieyan, S., Cianfrocco, M.A., amd W.O. Hancock. 2022. Kinesin-1, -2, and -3 motors use family-specific mechanochemical strategies to effectively compete with dynein during bidirectional transport. *Elife.* 11:e82228.
2. Arpağ, G., Norris, S.R., Mousavi, S.I., Soppina, V., Verhey, K.J., Hancock, W.O., and E. Tüzel. 2019. Motor Dynamics Underlying Cargo Transport by Pairs of Kinesin-1 and Kinesin-3 Motors. *Biophys J.* 116(6):1115-1126.
3. Arpağ, G., Shastry, S., Hancock, W.O., and E. Tüzel. 2014. Transport by populations of fast and slow kinesins uncovers novel family-dependent motor characteristics important for in vivo function. *Biophys J.* 107(8):1896-1904.
4. Feng, Q., Mickolajczyk, K.J., Chen, G.Y., and W.O. Hancock. 2018. Motor Reattachment Kinetics Play a Dominant Role in Multimotor-Driven Cargo Transport. *Biophys J.* 114(2):400-409.
5. Schroeder, H.W. 3rd, Hendricks, A.G., Ikeda, K., Shuman, H., Rodionov, V., Ikebe, M., Goldman, Y.E., and E.L. Holzbaur. 2012. Force-dependent detachment of kinesin-2 biases track switching at cytoskeletal filament intersections. *Biophys J.* 103(1):48-58.
6. Soppina, P., Patel, N., Shewale, D.J., Rai, A., Sivaramakrishnan, S., Naik, P.K., and V. Soppina. 2022. Kinesin-3 motors are fine-tuned at the molecular level to endow distinct mechanical outputs. *BMC Biol.* 20(1):177.
7. Schlager, M.A., Hoang, H.T., Urnavicius, L., Bullock, S.L., and A.P. Carter. 2014. In vitro reconstitution of a highly processive recombinant human dynein complex. *EMBO J.* 33(17):1855-68.
8. Urnavicius, L., Lau, C.K., Elshenawy, M.M., Morales-Rios, E., Motz, C., Yildiz, A., and A.P. Carter. 2018. Cryo-EM shows how dynactin recruits two dyneins for faster movement. *Nature.* 554(7691):202-206.
9. Ohashi, K.G., Han, L., Mentley, B., Wang, J., Fricks, J., and W.O. Hancock. 2019. Load-dependent detachment kinetics plays a key role in bidirectional cargo transport by kinesin and dynein. *Traffic.* 20(4):284-294.
10. Müller, M.J., Klumpp, S., and R. Lipowsky. 2008. Tug-of-war as a cooperative mechanism for bidirectional cargo transport by molecular motors. *Proc Natl Acad Sci U S A.* 105(12):4609-14.
11. Belyy, V., Schlager, M.A., Foster, H., Reimer, A.E., Carter, A.P., and A. Yildiz. 2016. The mammalian dynein-dynactin complex is a strong opponent to kinesin in a tug-of-war competition. *Nat Cell Biol.* 18(9):1018-24.

12. Elshenawy, M.M., J.T. Canty, L. Oster, L.S. Ferro, Z. Zhou, S.C. Blanchard, and A. Yildiz. (2019). Cargo adaptors regulate stepping and force generation of mammalian dynein-dynactin. *Nat Chem Biol.* 15(11):1093-1101
13. Hoeprich, G.J., Thompson, A.R., McVicker, D.P., Hancock, W.O., and C.L. Berger. 2014. Kinesin's neck-linker determines its ability to navigate obstacles on the microtubule surface. *Biophys J.* 106(8):1691-700.
14. Stern, J.L., Lessard, D.V., Hoeprich, G.J., Morfini, G.A., and C.L. Berger. 2017. Phosphoregulation of Tau modulates inhibition of kinesin-1 motility. *Mol Biol Cell.* 28(8):1079-1087.
15. Lessard, D.V., and C.L. Berger. The Microtubule Associated Protein Tau Regulates KIF1A Pausing Behavior and Motility. bioRxiv. 2021.08.11.455914, https://www.biorxiv.org/content/10.1101/2021.08.11.455914v1 (preprint posted August 11, 2021).
16. Chaudhary, A.R., F. Berger, C.L. Berger, and A.G. Hendricks. (2018). Tau directs intracellular trafficking by regulating the forces exerted by kinesin and dynein teams. *Traffic.* 19(2):111-121.
17. Chen, Y., N.C. Deffenbaugh, C.T. Anderson, and W.O. Hancock. (2014). Molecular counting by photobleaching in protein complexes with many subunits: best practices and application to the cellulose synthesis complex. *Mol Biol Cell.* 25(22):3630-42.
